# Human Engineered Heart Tissue as a Versatile Tool in Basic Research and Preclinical Toxicology

**DOI:** 10.1371/journal.pone.0026397

**Published:** 2011-10-20

**Authors:** Sebastian Schaaf, Aya Shibamiya, Marco Mewe, Alexandra Eder, Andrea Stöhr, Marc N. Hirt, Thomas Rau, Wolfram-Hubertus Zimmermann, Lenard Conradi, Thomas Eschenhagen, Arne Hansen

**Affiliations:** 1 Department of Experimental Pharmacology and Toxicology, Cardiovascular Research Center, University Medical Center Hamburg-Eppendorf, Hamburg, Germany; 2 Department of Pharmacology for Pharmacists, Cardiovascular Research Center, University Medical Center Hamburg-Eppendorf, Hamburg, Germany; 3 Department of Cardiovascular Surgery, Cardiovascular Research Center, University Medical Center Hamburg-Eppendorf, Hamburg, Germany; Cardiovascular Research Institute Maastricht, Maastricht University, The Netherlands

## Abstract

Human embryonic stem cell (hESC) progenies hold great promise as surrogates for human primary cells, particularly if the latter are not available as in the case of cardiomyocytes. However, high content experimental platforms are lacking that allow the function of hESC-derived cardiomyocytes to be studied under relatively physiological and standardized conditions. Here we describe a simple and robust protocol for the generation of fibrin-based human engineered heart tissue (hEHT) in a 24-well format using an unselected population of differentiated human embryonic stem cells containing 30–40% α-actinin-positive cardiac myocytes. Human EHTs started to show coherent contractions 5–10 days after casting, reached regular (mean 0.5 Hz) and strong (mean 100 µN) contractions for up to 8 weeks. They displayed a dense network of longitudinally oriented, interconnected and cross-striated cardiomyocytes. Spontaneous hEHT contractions were analyzed by automated video-optical recording and showed chronotropic responses to calcium and the β-adrenergic agonist isoprenaline. The proarrhythmic compounds E-4031, quinidine, procainamide, cisapride, and sertindole exerted robust, concentration-dependent and reversible decreases in relaxation velocity and irregular beating at concentrations that recapitulate findings in hERG channel assays. In conclusion this study establishes hEHT as a simple *in vitro* model for heart research.

## Introduction

Human embryonic stem cells (hESC) are pluripotent cells and can differentiate into all cell types of the body. Progenies of hESCs could become very useful tools for drug development, toxicology and therapeutic applications. Among hESC derivatives cardiomyocytes are of particular interest since electrophysiological properties of cardiomyocytes from laboratory animals such as mice, rats and swine are substantially different from human cardiomyocytes, limiting the validity of the obtained results. Despite the theoretical advantages, the use of hESC-derived cardiomyocytes is still hampered by inefficient differentiation, poor levels of maturation and the lack of experimental systems that would allow measurements of contractile function under defined conditions.

Cardiac differentiation of hESCs was achieved by undirected differentiation in serum containing media yielding 8% of beating embryoid bodies (EBs) [1]. Protocols relying on directed differentiation strategies resulted in higher cardiomyocytes yield. These include EB-based differentiation by growth factor cocktails mimicking early embryonic development, paracrine effects of END-2 cells via co-culture, conditioned media or a derived chemically defined media or the differentiation of confluent layers of hESCs by activin A and BMP4 under serum-free conditions [2]–[6]. Cardiomyocytes derived from these protocols appeared morphologically and functionally immature. Histologically, cardiomyocytes presented as polygonal to round cells with uneven cellular distribution of sarcomeres. Sarcomeric organisation of myofibrils was present predominantly in the perinuclear zone, but not in the periphery of the cell. Cellular alignment and uniform orientation were lacking. The immature histological phenotype was supported by electrophysiological characteristics. Action potential amplitude, maximal diastolic potential, upstroke velocity and duration suggested that hESC-derived cardiomyocytes resemble cardiomyocytes from 16 week old fetal hearts [3]. Previous strategies on the generation of hESC-CM based myocardial tissue engineering included the generation of matrix-based [7] as well as scaffold-free protocols [8], [9]. These protocols resulted in cardiac patches with a network of cardiomyocytes. The implementation of mechanical load resulted in improved alignment and maturation of cardiomyocytes and was accompanied with better survival after transplantation [10].

Torsades de pointes tachycardia (TdP) and ventricular tachycardia (VT) are frequent presentations of proarrhythmic side effects of various drugs. An important precondition for these tachycardias is prolongation of the QT interval in the ECG. This drug induced long QT Syndrome (LQT) develops when delayed rectifier potassium currents are inhibited. Delayed rectifier potassium currents are subdivided into a rapid (I*_Kr_*) and a slow component (I*_Ks_*). In human cardiomyocytes I*_Kr_* is the predominant form and hERG channels contribute substantially to this current. Many proarrhythmic drugs block hERG channels. Preclinical cardiac toxicity test panels typically include measurements of hERG channel activity in HEK293 cells, action potential duration in dog or rabbit Purkinje fibers and QTc interval and rhythm in instrumented dogs. These tests are recommended by the US Food and Drug Administration (FDA) and the European Medicines Agency (EMA), but remain partially insufficient because they are based either on non-cardiomyocytes or non-human cells, are difficult to standardize or unsuitable for large scale screening purposes [11], [12]. The problem is relevant since several approved drugs had to be withdrawn from the market because proarrhythmic side effects were recognized only in clinical use. Microelectrode arrays (MEA) have been proposed as a format for hESC-derived cardiomyocytes based toxicity tests, a technique which relies on the concept that beating clusters of cardiomyocytes generate external field potentials. Field potential durations are directly linked to action potential durations and are easy to define and measure [13]. HESC-derived cardiomyocytes displayed broadened field potentials in the presence of proarrhythmic compounds in a concentration-dependent manner [14], [13]. IC_50_ values in MEA were discrepant to findings in hERG channel assays, possibly due to cellular immaturity and technical limitations.

Engineered heart tissues (EHTs) are three dimensional force-generating cardiac tissue-like structures. EHTs from neonatal rat hearts develop a high degree of cellular differentiation, longitudinal orientation, intercellular coupling and force generation [15], [16]. The principal usefulness of these techniques for *in vitro* testing [17], [18] and cardiac repair [19] has been demonstrated. Miniaturisation and automation has been accomplished with strip-format fibrin-based mini-EHTs. This format proved suitable to detect proarrhythmic effects of drugs in neonatal rat heart EHT [20]. The present study aimed at establishing a robust protocol for the generation of hESC-derived EHT and determining its validity in detecting effects of proarrhythmic compounds.

## Results

### Cardiomyocyte differentiation and EHT generation

**Figure 1:**
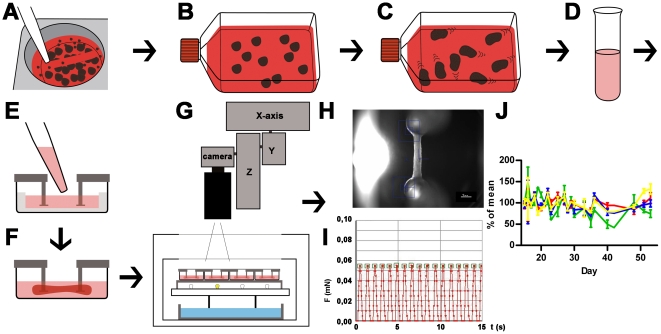
Schematic illustration of cardiac differentiation, EHT generation and analysis. A: Undifferentiated hESC colonies are detached after collagenase treatment with 5 ml pipette; B: EBs are formed in ultra-low attachment flasks (for details see Figure S1). C: Cardiomyocytes are differentiated and onset of beating in EBs is monitored; D: EBs are enzymatically dissociated into single cells and a mastermix (cells, fibrinogen, thrombin) is prepared; E: Mastermix is pipetted into EHT casting molds; F: Within two weeks coherent beating EHTs develop under cell culture conditions between silicone posts; G: EHTs in 24 well plates are analyzed by automated video-optical recording, therefore a camera is placed above the incubator and directed to each well by x-y-z-coordinate motor system; H: Movies are recorded, automated figure recognition modus identifies top and bottom end of each EHT (blue squares); I: Based on post deflection, post geometry and elastic propensity force development is calculated and recorded over time. Contraction peaks are automatically recognized (green squares) and parameters of contraction are calculated and summarized in a report; J: Force (red), frequency (green), contraction velocity (blue), relaxation velocity (yellow) of 4 EHTs between day 15 and day 55 of development, lines show means ± SEM.

**Figure 2:**
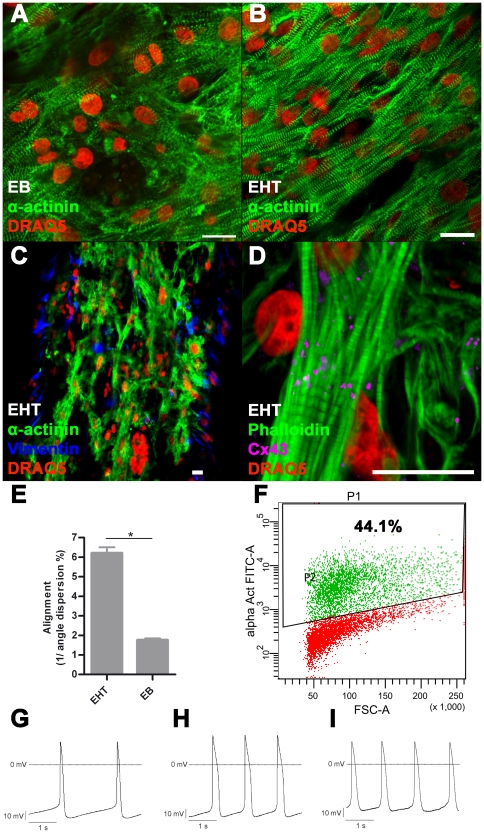
Immunofluorescence, FACS, electrophysiology. A: Immunofluorescence staining of 2–3 week old EB, B–D: Immunofluorescence staining of EHT, scale bar 20 µm; E: Quantification of alignment: In EHT format cardiomyocytes are significantly more aligned than in EB-format *P<0.05 (Student's *t*-test), bars show mean ± SD. F: Representative FACS analysis of dissociated EBs (2–3 weeks) before EHT generation, P1: All gated cells, P2: α-actinin positive cells. Representative recordings of APs: G: EBs (2–3 weeks), H: EBs (7–8 weeks), I: EHT (5 weeks): 7–8 week old EBs as well as EHT derived cardiomyocytes have longer APSs compared to young EB derived cardiomyocytes. Furthermore the maximal diastolic potential is particularly low in EHT derived cardiomyocytes.

Cardiomyocyte differentiation was performed with a modified EB-based, three-stage, growth factor cocktail protocol [2]. Modifications of the published protocol included the expansion of hESCs (hES2) on Matrigel® with conditioned medium, allowing formation of EBs without feeder depletion (Figure 1). EB formation was performed in conditioned medium. Differentiation was induced in RPMI/B27 in the presence of the Rho kinase inhibitor Y-27632 (10 µM) and HEPES (10 mM) (see Figure S1). The duration of mesodermal induction by BMP-4, activin-A and basic FGF had impact on cardiomyocyte differentiation. The optimal duration appeared to depend on the quality of input hESCs which can not be monitored in real time. In our hands insufficient differentiation outcomes were improved after readjusting the duration of mesodermal induction (24–72 hours). Onset of beating occurred between day 10 and day 15. The number of beating EBs increased after transfer to serum-containing media and switch to 20% oxygen and reached 60–90% (Movie S1). A 100 cm^2^ culture of undifferentiated hESCs (∼15×10^6^) resulted in approximately 1.5×10^6^ differentiated cells out of which 41±10% (n = 3) stained positive for α-actinin in FACS analysis (Figure 2F).

Differentiated EBs were enzymatically dispersed. The dissociated cells were mixed with fibrinogen and thrombin and poured into slit-formed agarose casting molds in a standard 24-well plate in which two elastic silicone posts per well were inserted from above (600,000 cells/EHT; Figure 1D, E). After fibrin polymerisation (2 hours) silicone racks with 4 pairs of silicone posts each and the respective cell-fibrin gel were transferred to new 24 well plates and maintained under standard cell culture conditions for two weeks (Figure 1F). Onset of coherent, silicone post-deflecting beating activity occurred on day 5–10. Contractile EHTs were subjected to automated video-optical recording and analysis between day 15–55 (Figure 1G–I). Human EHTs (hEHT) showed stable values for force, frequency, contraction and relaxation velocity for several weeks (Figure 1J) allowing for repeated measurements after washout. The spontaneous beating pattern was regular with frequencies ranging from 40–70 beats per minute and average force development of 0.061±0.013 mN (n = 4). Based on the simplifying assumption that the EHTs are circular cylinders and a mean diameter of 0.72 mm this is equivalent to 0.12 mN/mm^2^ (Movie S2, information S1).

### Transcript analysis

**Figure 3:**
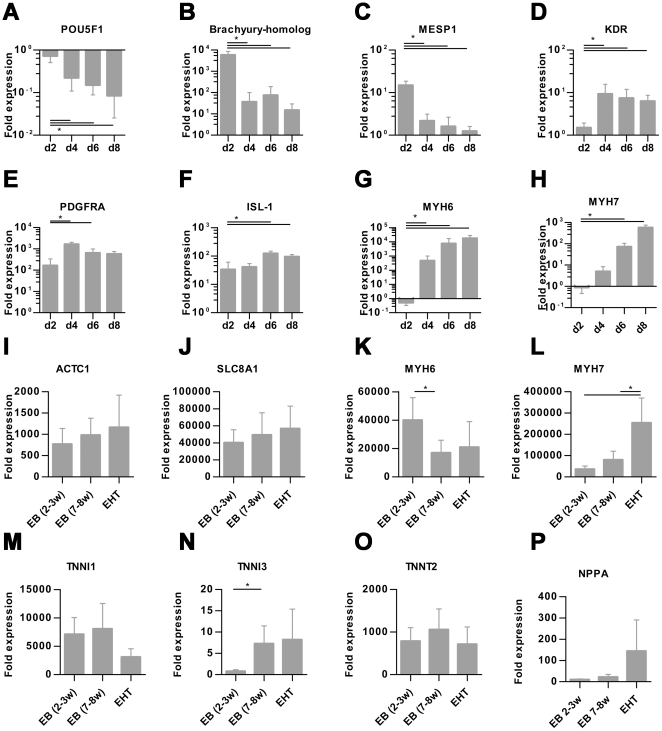
Transcript analysis. A–H: Quantitative RT-PCR analysis of stemness marker (*POU5F1*, POU class 5 homeobox 1), mesodermal marker (*brachyury homolog*, *MESP1*, mesoderm posterior 1 homolog, *KDR*, vascular endothelial growth factor receptor 2, *PDGFRA*, platelet derived growth factor receptor alpha) and cardiac marker (*ISL1*, Islet-1, *MYH6*, α-myosin heavy chain, MYH7, ß-myosin heavy chain,) in EB-based cardiac differentiation on day 2, 4, 6, and 8, normalized to undifferentiated hESCs. For these experiments Stage 1 duration (mesodermal induction) was set to 24 hours, 4–7 biological replicas. I–L: Quantitative RT-PCR analysis of alpha actin *(ACTC1)*, sodium/calcium exchanger *(SLC8A1)*, *MYH6* and *MYH7* in EBs (2–3 weeks old), EBs (7–8 weeks old) and EHTs (5 weeks old) normalized to undifferentiated hESC, 4–6 biological replica. Bars show mean ± SD, *P<0.05 (Student's *t*-test), bars show means ± SD.

Cardiac differentiation was monitored by analyzing transcript levels of marker genes. The stemness marker POU class 5 homeobox 1 (*POU5F1*, *OCT4*) showed highest expression in undifferentiated hESCs and a sharp decline in mRNA levels upon differentiation. The mesodermal markers brachyury homolog (*T*), mesoderm posterior 1 homolog (*MESP1*), vascular endothelial growth factor receptor 2 (*KDR*) and platelet derived growth factor receptor alpha (*PDGFRA*) were significantly upregulated at early time points of differentiation (d2–d4) and remained significantly upregulated (*KDR*) or declined again (*T*, *MESP1*, *PDGFRA*). Islet-1 (*ISL1*) a progenitor marker of the development of the secondary heart field [21] was significantly upregulated on day 6 and 8. Transcripts of the cardiac markers α-myosin heavy chain (α-MHC, *MYH6*, fast isoform) and ß-myosin heavy chain (ß-MHC, *MYH7*, slow isoform) were essentially absent on day 2 of the differentiation protocol and dramatically increased between day 4 and 8. Comparison of the level of upregulation on day 8 revealed that α-myosin heavy chain (10,000-fold upregulation) was the dominant form and ß-myosin heavy chain expression (500-fold) substantially lower (Figure 3A–H).

To evaluate whether the EHT format had an effect on cardiomyocyte maturation when compared to the standard EB format, cardiac markers were analyzed in 2–3 week old EBs, 7–8 week old EBs and hEHTs that had been cultured for 5 weeks after having been generated from 2–3 week old EBs (to age-match 7–8 week old EBs). This analysis (n = 4–6 independent experiments) revealed that alpha actin (*ACTC1*), sodium/calcium exchanger, (*SLC8A1*), slow skeletal troponin (*TNNI1*), troponin I Type 3 (*TNNI3*), troponin T (*TNNT2*), and α-MHC transcript levels did not differ between the different culture formats, while ß-MHC expression was upregulated two- to threefold stronger in the EHT format. The mRNA levels of atrial natriuretic peptide (*NPPA*) also tended to be higher in EHTs, but the difference did not reach significance (Figure 3I–P).

To determine the character of the non-myocyte cell population transcript concentrations were analyzed in 2–3 and 7–8 week old EBs as well as 5 week old EHTs (n = 4–5 independent experiments) by quantitative PCR with primers for neuroectodermal (*NEUROD1*, neurogenic differentiation 1; *SOX1* sex determining region Y-box 1), endodermal (*FOXA2*, forkhead box A2; *FOXA3*, forkhead box A3), hematopoietic (*PTPRC*, *CD45*, protein tyrosine phosphatase, receptor type, C; *GATA1* globin transcription factor 1), endothelial (*CDH5*, VE-cadherin; *PECAM1*, *CD31*, platelet/endothelial cell adhesion molecule), mesodermal somite (*MEOX1*, mesenchyme homeobox 1) and (myo)fibroblast (*CNN1*, calponin; *ACTA2*, smooth muscle actin) development (Figure S2) [2]. *CD45* and *GATA1* were undetectable in all samples. *CNN1*, *CDH5*, *PECAM1*, *FOXA2* and *NEUROD1* were expressed at low levels (∼10-fold of undifferentiated hESC) and showed no significant differences between the three groups (Figure S2). *ACTA2* was >100-fold upregulated in 2–3 week-old EB compared to undifferentiated hESC, and this increase continued with time of cultivation and even more so in the EHT format, reaching ∼600-fold. *MEOX1* and *SOX1* showed essentially a mirrored pattern with relative high levels in 2–3 week-old EBs (>10-fold undifferentiated hESC), a decline over time and lowest levels in EHTs (Figure S2). *FOXA3* was expressed at very low levels (∼0.5 fold undifferentiated hESC), which further decreased significantly in the EHT format.

### Immunofluorescence

Immunofluorescence staining of EBs revealed α-actinin-positive cells with partial sarcomeric organisation (Figure 2A). Cardiomyocytes showed random orientation and poor alignment. In contrast, cardiomyocytes in EHT showed enhanced alignment and predominant longitudinal orientation along force lines (Figure 2B). Quantification by blinded angle dispersion analysis revealed significantly higher alignment in EHTs (Figure 2E, *P<0.05, Student's *t*-test). Sarcomeric organisation was evenly distributed and well developed, both around the nuclei and the periphery of the cells. Cardiomyocytes showed expression of connexin-43, but not confined to intercalated disks as in the adult heart (Figure 2D). This pattern is similar to connexin-43 expression in rat EHT [20]. EHT also contained α-actinin-negative cells reflecting the mixed population of input cells. Immunofluorescence staining for vimentin, which identified a subpopulation of these along the surface of the EHT, indicates that they are fibroblast-like cells (Figure 2C).

### Electrophysiology

To compare electrophysiological properties of cardiomyocytes in the EB format and inside hEHT, cells were dissociated by mechanical means and impaled by sharp electrodes. All hESC-derived cardiomyocytes displayed spontaneous action potentials (AP) synchronous to their contractions. Basic AP parameters of hESC-derived cardiomyocyte cultures at different stages and culture conditions, respectively, are summarized in Table 1. Cells exhibited discontinuous AP firing patterns with frequencies ranging from 0.2 to 1.3 Hz in EBs and 0.08 to 0.7 Hz in EHTs. Higher firing rates were accompanied by shorter AP durations (APD). Overall, cardiomyocyte APs fulfilled the criteria adopted for classification as primitive or nodal-like [22]. 7–8 week old EBs featured higher APD_80_ and upstroke velocity than 2–3 week-old EBs, which argues for increasing maturation of hESC over time (Figure 2G–I, Figure S3). Mean APD_80_ of cardiomyocytes isolated from EHTs was approximately twice as long as APD_80_ in EB-derived cardiomyocytes. However, AP duration varied greatly within the EHTs, ranging from 260 to 1200 ms. Cardiomyocytes with longer APDs were characterized by greater AP upstroke velocity. Maximal diastolic potentials were low in 2–3 week old EBs (average −58.2 mV) and extended periods in EB format did not lead to maturation (7–8 week old EBs: average −60.7 mV). The maximal diastolic potential in EHTs was even less negative (average −49.1 mV) and this was consistent between the subpopulations with different APDs. In all spontaneously beating hESC cultures, exposure to nisoldipine (1 µM) completely interrupted electrical activity as well as contractions, indicative of a crucial contribution of transsarcolemmal calcium influx through L-type calcium channels to spontaneous diastolic depolarization and AP generation (example of hEHT shown in Figure S3). Application of the sodium channel blocker tetrodotoxin (TTX, 3 µM) induced a significant decrease in AP upstroke velocity, accompanied by a significant prolongation of APD_80_ and diastolic interval (P<0.05, Student's *t*-test, *n = 5* for each parameter and hESC culture). The effects of TTX were particularly pronounced in 7–8 week old EBs (Figure S3), suggesting that a slowly inactivating component of the sodium current (referred to as late I_Na_) contributes to the AP firing in hESCs. Finally, exposure to E-4031 (300 nM) induced a significant prolongation of APD in hESC-derived cardiomyocytes (example of 7–8 week-old EBs shown in Figure S3; P<0.05, Student's *t*-test, *n = 4*).

**Table 1:** Properties of spontaneous APs of hESC-derived cardiomyocytes.

	APD_80_ (ms)	MDP (mV)	APA (mV)	dV/dt_max_ (V/s)
EB (2–3 w; *n* = 14)	268±14	−58±1.0	82±3.4	8.2±0.6
EB (7–8 w; *n* = 13)	384±33*	−61±0.7	88±2.0	12.2±1.0*
EHT (short APD, *n* = 7)	341±23*	−49±1.6*	66±3.6*	5.6±0.7*
EHT (long APD, *n* = 7)	887±71*	−49±1.2*	85±2.8	10.4±1.2

Data are presented as means ± SEM, with *n* representing the number of cells from 2–3 or 7–8 week-old EB cell aggregates or from EHT samples (cultured for 5 weeks after generation from 2–3 week-old EBs). Cut-off for short versus long APD was 500 ms. Student's two-tailed paired or unpaired *t*-test as appropriate was used to assess statistical significance versus EB (2–3 w), indicated by *P<0.05 (Student's *t*-test).

### Pharmacology of hEHT

**Figure 4:**
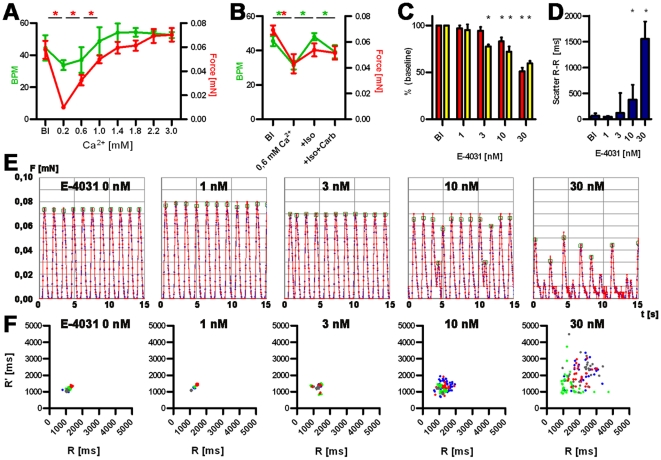
Functional analysis of hEHTs. A and B: Calcium concentration-response curve: Development of force of contraction (red, mN) and frequency (green, beat per minute, BPM) and baseline (Bl) and increasing Ca^2+^ concentration (A), Response to isoprenaline and carbachol (B). C: Analysis of force and relaxation velocity in the presence of E-4031. Red: force, yellow: relaxation velocity, depicted as percent of baseline values. *P<0.05 (Student's *t*-test), 4 biological replica, bars show means ± SD. D: Scatter of beat-to-beat variability in the presence of E-4031, *P<0.05 (Mann-Whitney U test), 4 biological replica, bars show median ± interquartile range. E: Original recordings (15 sec each) of spontaneous EHT contractions under increasing concentrations of E-4031 (0–30 nM). F: Statistical evaluation of beat-to-beat variability of the experiments depicted under E. Ordinates indicates the distance from a given twitch to the following, the abscissa the distance to the previous twitch. Biological replicas are discriminated by color code.

Calcium concentration-response curves showed that force development in hEHT strongly depended on extracellular calcium concentrations (Figure 4A, B). Lowering calcium from 1.8 mM (baseline) to 0.2 mM decreased force by 90%. Cumulative increases in calcium concentrations increased force until a plateau was reached between 2.2 and 3.0 mM. In the presence of 0.6 mM calcium the β-adrenergic agonist isoprenaline (100 nM) had a positive chronotropic effect, which was accompanied by a trend towards an increase in force (not significant), both were reversed by the muscarinic receptor agonist carbachol (10 µM, Figure 4B).

To determine the suitability of hEHT to detect proarrhythmic effects of drugs, hEHT were exposed to the known proarrhythmic compounds E-4031, procainamide, cisapride, quinidine, and sertindole. Concentrations were chosen according to published IC_50_ in hERG assays [23], [24]. The antibiotic ampicillin served as negative control. Proarrhythmic compounds led to a concentration-dependent reduction in relaxation velocity, decline in contractile force and irregular beating patterns (Figure 4, Table 2, information S1). Maximal effects amounted to reductions by 30–50%. In contrast, no concentration-dependent changes were monitored in the presence of even high concentrations of ampicillin (1 mM). We have shown previously that time and rate of relaxation were the primary parameters affected by proarrhythmic compounds in rat EHTs, likely as a surrogate of action potential duration [20]. Based on these observations, the concentration of each respective compound was determined that led to a significant decrease in relaxation velocity in hEHTs. E-4031 is a potent and specific inhibitor of I*_Kr_* and, at 3 nM, led to an exclusive decrease of relaxation velocity. This matches well with hERG channel assay results (IC_50_ 7 nM). At a higher concentration (30 nM) force of contraction and contraction velocity declined significantly as well (Table 2). Other proarrhythmic compounds are less specific hERG channel blockers and additionally modulate sodium channels (quinidine, procainamide), calcium channels (quinidine), adrenergic (sertindole), cholinergic (quinidine), dopaminergic (sertindole) and 5-HT receptors (sertindole, cisapride). However, all compounds decreased relaxation velocity at concentrations close to the reported IC_50_ values in hERG channel assays (Table 2). Moreover, all proarrhythmic compounds resulted in irregular beating patterns at similar concentrations (Table 2, information S1).

**Table 2:** Comparison of threshold concentrations.

	Relaxation velocity	RR-scatter	Published IC_50_ (hERG)
E-4031	3 nM	10 nM	7.7 nM
Procainamide	100 µM	100 µM	139 µM
Sertindole	10 nM	300 nM	14 nM
Quinidine	1000 nM	1000 nM	490 nM
Cisapride	30 nM	30 nM	9.4 nM
Ampicillin	-	-	-

Comparison of the threshold concentrations at which the compounds significantly decreased relaxation velocity (*P*<0.05 vs baseline, Student *t*-test) or induced irregular beating patterns (RR-scatter, *P*<0.05 vs baseline, Mann-Whitney U Test) with published IC_50_ values in hERG channel assays [24] [23]. RR-scatter was defined as the interdecile range of beat-to-beat interval length (information S1).

## Discussion

In this manuscript we report a new method to generate 3D, force-generating human heart muscles (hEHT) from hESC that are amenable to automated drug screening in a 24-well format. Important aspects are (1) the use of an unpurified population of differentiated hESC with 40% cardiomyocytes, (2) the relative simplicity of the protocol, (3) the high degree of 3D cardiac tissue-like structure of hEHT, (4) the stability and regularity of spontaneous beating for weeks allowing repeated measurements, and (5) the high sensitivity towards proarrhythmic compounds. As such the method may be useful for predictive toxicology. Furthermore, it represents an important step towards iPS-mediated disease modelling and, for the first time, opens a realistic opportunity to study the effect of overexpressing or knocking down proteins of interest in human cardiac muscle tissue.

One of the current limitations of hESC-derived cardiac myocytes is their immature and unorganized phenotype in the EB format or 2D cultures [3], [5]. Three ways were explored to analyze whether the hEHT format improves maturation of cardiomyocytes. Histologically, cardiomyocytes in EHTs showed substantially better sarcomeric organisation and alignment than cardiomyocytes in EBs and other published culture formats. Likely reasons are the straight force lines imposed by spanning of the tissue between the two silicone posts and the fact that hEHTs continuously perform contractile work against the elastic posts as previously described in neonatal rat EHTs [20]. Quantitative RT-PCR provided some evidence that the EHT format is promoting maturation: Transcript concentrations of β-MHC, the adult isoform in humans, increased over time significantly only in EHT format but not in EB format, similar to what has been described for the transition of immature to adult human ventricular cardiomyocytes [25]. Somewhat in contrast to these findings were the electrophysiological characteristics. On the one hand, ventricular cardiomyocyte-like APD supported maturation. On the other hand, hEHT-derived cardiomyocytes showed slow upstroke velocities and low MDP. The latter is a characteristic feature of hESC-derived cardiac myocytes and has been explained by insufficient I*_K1_* current [3]. It remains unclear why cardiomyocytes isolated from EHTs had even lower MDPs than those from EBs. Technical reasons (e.g. consequences of enzymatic dissociation) or the fibrin matrix may be one factor, but they cannot fully explain this finding since MDPs of cardiomyocytes from neonatal rat EHTs amounted to −79 mV under similar experimental conditions [20]. Thus, cardiac myocytes in hEHT, despite excellent structural maturity, appear to be electrophysiologically immature. This immaturity likely contributes to very regular and stable beating over weeks, which differs from (the likely more mature) rat EHTs. The non-myocyte content in EHTs was analyzed and revealed that the contribution of derivatives of neuroectodermal and endodermal as well as other mesodermal lineages (hematopoietic, endothelial, mesodermal somite) was very small after cardiac differentiation and EHT generation. The strong upregulation of ACTA2 transcripts and the immunofluorescent staining of vimentin-positive cells in the EHTs indicate that (myo)fibroblast was the predominant non-myocyte subpopulation.

Our automated analysis system is based on video-optical recordings of spontaneously beating hEHTs. Thus, drug effects by definition are restricted to affections of contractile pattern. The parameters are frequency of contraction (bpm), force of contraction (mN), times of contraction/relaxation (ms), maximal velocity of contraction/relaxation (dF/dt, mN/sec) and onset of irregular beating pattern. All parameters were analyzed in the presence of increasing concentrations of extracellular calcium, isoprenaline ± carbachol or proarrhythmic compounds. Experiments with calcium, isoprenaline and carbachol demonstrated that hEHT responded to standard inotropic interventions similar to human heart muscle. Importantly, the calcium-response curve was in a relatively normal range (maximal effect reached at 3 mM), in this respect they differ from rat EHT which respond to unusually low calcium concentrations and reach their maximum at 1.6 mM [16]. The responses to isoprenaline and carbachol, albeit relatively small in size compared to adult human heart, indicate the principal existence of an adrenergic and muscarinic receptor system.

Experiments with the specific I*_Kr_* blocker E-4031 and several other compounds, which share hERG blockade and proarrhythmic effects in humans as a common feature, showed significant decreases in repolarisation velocity and the occurrence of irregular beating pattern (scatter of beat-to-beat variability). Threshold concentrations for these parameters compare well with published IC_50_ values determined in hERG channel assays. This suggests that these two parameters are the best surrogate of hERG inhibition and proarrhythmic potential in hEHTs. Interestingly, the antipsychotic drug sertindole prolonged relaxation at the hERG concentration of 10 nM, but induced significant beat-to-beat variability only at a 30-fold higher concentration. The exact reason is presently unknown, but likely pharmacological properties in addition to hERG-inhibition account for it and can only be distinguished by studying larger panels of indicator compounds. High concentrations of proarrhythmic compounds led to a decline in force of spontaneous contractions. This is likely the consequence of repolarisation inhibition (I*_Kr_* block) on top of insufficient I*_K1_* current, resulting in a reversible depolarisation block. In this context the decrease in contractile force can not be interpreted as a typical negative inotropic effect as exerted for example by L-type calcium channel blockers. In addition the expected positive inotropic effect of repolarisation inhibitors (by prolongation of plateau phase and concomitant increase in calcium influx) is masked in this system, possibly because maximal force development is already almost reached at the standard calcium concentration in the medium of 2 mM (see Figure 4A).

Compared to patch clamp experiments, video-optical recordings and analysis of hEHTs are simple and require only adjustment of XYZ-coordinates of the camera for each hEHT. Recording of movies, figure recognition, analysis of contractility, graphical presentation and calculation of averaged contraction parameters are determined in an automated mode. Importantly and in contrast to most other assays, this test system utilizes human cardiomyocytes and thus integrates the complexity of this human cell type, moreover in a 3D tissue structure. HEHTs can be used repeatedly after over night wash-out periods.

A number of limitations of this protocol need to be stated. The first one relates to the still limited number of differentiated cardiomyocytes from hESC and their immaturity. The differentiation protocol is time consuming and requires continuous monitoring. It is working well with the hES2 cell line, but has not been optimized for other hESC or human iPS cell lines yet. At present, 2–4 EHTs can be generated per differentiation of 100 cm^2^ of undifferentiated hESCs, which translates into approximately 50 € per hEHT for media and supplements. An important reason is the low cardiomyocytes/input of undifferentiated hESC-ratio (0.04/1) which is inferior to ratios of other published statagies: 3/1 [5] 0.8/1 [6]. EHT-derived cardiomyocytes have significantly lower maximal diastolic potentials than ventricular cardiomyocytes and can therefore not be considered as an *in vitro* model of mature myocardium. Recent studies have highlighted the role of NRG-1ß/Erbß signalling in the specification of cardiomyocytes subtypes of differentiating hESCs [26] and might therefore be useful in the process of maturation. The second limitation is the lack of direct measurements of APD and/or calcium transients. We are currently setting up a suitable recording system which aims at monitoring of calcium transients (and APs) with fluorescent dyes and also allows for electrical pacing and continuous perfusion. Finally, the true predictive value of this method for toxicology studies in addition or as an alternative to current tests needs to be assessed with larger series of known proarrhythmic and safe drugs.

Overall hEHTs show important features of human myocardium, recapitulate findings in conventional toxicity tests and might be a useful *in vitro* surrogate in the context of predictive toxicology, drug development and disease modelling.

## Materials and Methods

### Culturing undifferentiated hESC

HES2 cells (HES2.R26 [27]) were propagated on Matrigel® with CF1-MEF conditioned medium according to published protocols [28].

### Cardiomyocytes differentiation

Confluent layers of hESC colonies were digested with collagenase IV (Gibco 17104, 1 mg/ml, 1 ml/10 cm^2^) until edges of the colonies start to dislodge (10–20 minutes). Collagenase was removed and washed with 2 ml PBS/10 cm^2^. CF1-MEF conditioned medium was added (1 ml/10 cm^2^). Embryoid bodies (EBs) were generated by carefully scraping off colony fragments with a 5 ml-pipette tip. Colony fragments were collected and remaining colonies were detached with a cell scraper. EB formation was performed in ultra low attachment cell culture flasks (ULA-CCF, Corning 3815), with colony fragments of 2.5 cm^2^ (undifferentiated hESC layer) per ml conditioned medium. Differentiation was performed in RPMI-B27 medium containing RPMI 1640 (Gibco 21875), B-27 supplement (2%, Gibco 0080085-SA), Penicillin/Streptomycin (0.5%, Gibco 15140) and HEPES (10 mM). Y-27632 (10 µM, Biaffin PKI-27632-010) and growth factors were added as specified (Figure S1).

After 24 hours EBs were collected in 50 ml tubes. ULA-CCFs were washed twice with PBS, solutions were added to the tubes and centrifuged (4 minutes, 300 rpm). Pelleted EBs were resuspendend in 20 ml mesodermal induction medium (stage I, basic FGF (5 ng/ml, R&D 233-FB), Activin-A (6 ng/ml, R&D 338-AC), BMP-4 (10 ng/ml, R&D 314-BP), Y-27632 (10 µM)) and transferred back into the ULA-CCF. After 1–3 days EBs were collected in 50 ml tubes. ULA-CCFs were washed twice with PBS, solutions were added to the tubes and centrifuged (4 minutes, 300 rpm). EBs were resuspendend in 15 ml cardiomyocyte induction medium (stage II, DKK-1 (150 ng/ml, R&D 5439-DK), VEGF (10 ng/ml, R&D 293-VE), Y-27632 (10 µM) and transferred back into the ULA-CCF. After 3 days EBs were collected in 50 ml tubes. ULA-CCFs were washed twice with PBS, solutions were added to the tubes and centrifuged (4 minutes, 300 rpm). EBs were resuspendend in 15 ml cardiomyocyte induction medium (stage III, DKK-1 (150 ng/ml), VEGF (10 ng/ml), basic FGF (5 ng/ml)). After 5–7 days 5 ml of RPMI-B27 medium was added every second day until the first beating EBs were detected. EB formation and differentiation was performed at 95% humidity, 37°C, 5% oxygen, 5% CO_2_. EBs were transferred to serum-containing medium (DMEM, Gibco 41965, 1% L-glutamine, 1% NEAA, 0.5% Penicillin/Streptomycin, 20% fetal bovine serum, 100 µM 2-mercaptoethanol) between day 12–15 and were kept at 20% oxygen. Analysis of contractility and dissociation for EHT generation was performed around day 15–20 (Movie S1).

### Total RNA preparation and quantification of gene expression

Total RNA was prepared with Qiagen RNeasy according to manufacturer's instructions. For reverse transcription and quantitative RT-PCR the High Capacity cDNA Reverse Transcription Kit (Applied Biosystems) and POWER SYBR® Green PCR Master Mix (Applied Biosystems) were used according to manufacturer's instructions. Experiments were performed on an ABI PRISM 7900HT Real-Time PCR system (Applied Biosystems). Quantification was performed by determining ΔΔCT analysis using glucoronidase (*GUSB*) as a housekeeping gene and undifferentiated hESCs as reference. PCR products were verified by amplicon size in agarose gels and melting curve analysis. Primer sequences are listed in Table S1.

### EHT generation

EBs were dissociated with 500 µl of a pre-warmed mixture (37°C) of collagenase and trypsin (approximately 1 mg/ml collagenase in 0.5% trypsin solution) for 2–5 minutes under continuous trituration with a 100 µl pipette. Dissociated cells were washed twice by adding 1 ml of serum containing media and centrifugation for 2 min at 200 g. After cell counting human EHTs were generated according to a protocol recently described for neonatal rat cardiomyocytes [20]. 0.6×10^6^ differentiated cells were used per EHT (100 µL). Human EHTs were maintained under cell culture conditions (37°C, 95% humidity, 40% oxygen) and medium was changed on Mondays, Wednesdays and Fridays. Medium composition was: DMEM (Biochrom F0415), 10% horse serum (Gibco 26050), 2% chick embryo extract, 1%, penicillin/streptomycin (Gibco 15140), insulin (10 µg/ml, Sigma-Aldrich I9278), tranexamic acid (400 µM, Sigma-Aldrich 857653) and aprotinin (33 µg/ml, Sigma-Aldrich A1153). Y-27632 (10 µM) was added to the medium for the first 24 hours. Silicone racks were custom made by Jäger Gummi und Kunststoff GmbH and Siltec GmbH. EHTs were subjected to further analysis after 5 weeks, thus matching the age of 7–8 week old EBs.

### FACS analysis

Embryoid bodies were harvested and dissociated into single cells as described above. Cells were fixed and permeabilized with BD Cytofix/Cytoperm™ solution (BD Biosciences 554722) for intracellular FACS, then incubated in blocking solution (TBS 0.05 M, pH 7.4, 10% FCS, 1% BSA, 0.5% Triton X-100). Staining was performed with anti-α-actinin (Sigma-Aldrich A7811, 1∶800) as primary antibody and Alexa fluor® 488 (goat anti-mouse 2 mg/ml, Molecular Probes A11017, 1∶400) as secondary antibody in antibody solution (TBS 0.05 M, pH 7.4, 1% BSA, 0.5% Triton X-100). Negative control was performed without primary antibody. Data were acquired on a BD FACSCanto II flow cytometer and analyzed using BD FACSDiva Software 6.0 (BD Biosciences).

### Action potential measurements

Action potentials (APs) were recorded from cells in spontaneously beating, partially dissociated EB cell clusters and EHTs with the conventional intracellular recording technique in the zero current clamp mode. All experiments were carried out at 35–37°C in serum-free DMEM (containing the following inorganic salts in mM: 110 NaCl, 5.4 KCl, 0.9 NaH_2_PO_4_, 0.8 MgSO_4_, 1.4 CaCl_2_, 44 NaHCO_3_) continuously gassed with carbogen to provide oxygenation and a pH of 7.4–7.6. The sharp microelectrodes had tip resistances ranging from 15 to 20 MΩ when filled with 3 M KCl. The pipette offset was compensated; the junction potential (1–2 mV) was negligible. About 20 hours before measurement, the EHTs were exposed to chymotrypsin (0.0025%, Sigma-Aldrich C4129) and collagenase CLS type II (0.2 mg/ml (65 units/ml); Worthington NY 4176) for 30 minutes to facilitate impalement of the muscle cells within the fibrin matrix. Data acquisition and analysis was achieved by the use of an EPC-9 patch clamp amplifier in combination with the Pulse/PulseFit 8.11 software (HEKA, Lamprecht, Germany). Output signals were digitized at 1 kHz. The following parameters were measured: AP duration at 80% repolarization (APD_80_) and AP amplitude (APA), maximum AP upstroke velocity (dV/dt_max_), and maximum diastolic potential (MDP). The pharmacological agents used in the electrophysiological studies included E-4031 (Eisai, Tokyo, Japan), tetrodotoxin (TTX, Alomone Labs, Jerusalem, Israel) and the L-type calcium channel inhibitor nisoldipine (Bayer AG, Leverkusen, Germany) at final concentrations of 0.3, 3 and 1 µmol/L, respectively.

### Concentration-response curves for calcium and pro-arrhythmic drugs

Calcium-concentration response curves were established in DMEM, 1% penicillin/streptomycin, 10 mM HEPES, and 100 µM acetylcysteine with calcium concentrations adjusted to the desired final concentration (0.2–3.0 mmol/L). Equilibration time was 20 minutes. Measurements were performed under spontaneous activity by automated video-optical recording and analysis (customized software, Consulting Team Machine Vision) as previously described [20]. Contractile force F was calculated from the degree of post deflection δ (µm), silicone post length (L) and radius (R) and elastic modulus (E) by the formula F = 3πER^4^δ/4L^3^ [29]. Contraction- and relaxation velocities were calculated as the average maximal steepness of twitch contraction or relaxation, respectively. Each value represents the mean of all measurable contraction twitches in the recording period of 60 seconds per hEHT.

Human EHTs exhibiting well detectable spontaneous post deflections were subjected to measurement of proarrhythmic drugs. EHTs were equilibrated in DMEM, 1% penicillin/streptomycin, 10 mM HEPES, 100 µM acetylcysteine for one hour. Compounds were dissolved in DMSO (E-4031, quinidine, procainamide, sertindole, cisapride, ampicillin) and added to the EHTs at cumulative concentrations 30 min before measurement. Isoprenaline and carbachol were used at 100 nM and 10 µM, respectively.

### Immunofluorescence

EBs/EHTs were fixed in 4% formalin over night. Blocking, antibody incubation and washing steps were performed with whole mount samples for 24 hours each. Antibodies and solutions were used as follows: α-actinin (Sigma A7811, 1∶800), connexin-43 (BD 610061, 1∶250), DRAQ5 (Biostatus BOS-889-001-R050, 1∶1000), vimentin (R&D MAB2105, 1∶50), phalloidin (Invitrogen A-12379, 1∶60), secondary antibodies: goat anti-mouse Alexa®488 (Invitrogen A-11017, 1∶800), goat-anti-mouse Alexa®546 (Invitrogen A-11003, 1∶800), goat anti-rat (R&D NL013, 1∶200). Confocal laser scanning microscopy was performed with Zeiss LSM 510 META system.

### Alignment quantification

To calculate alignment of cardiomyocytes in the EB- and EHT-format, a total number of 17 randomly chosen pictures (11 EHT, 6 EB) showing immunofluorescence staining of sarcomeric proteins (α-actinin, cTNT, phalloidin) was analyzed. Orientations (angles) of 10 to 20 prominent sarcomeric structures per picture were measured by four experimentators and dispersion of angles was calculated as interquartile range. Dispersion was expressed as percent (90°≙100%≙1). The alignment score was defined as the inverse value of relative angle dispersion [10]. Statistical difference were determined with *P<0.05 (Student's *t*-test). An averge angle dispersion of 9° (10%, 0.1) would result in an alignment value of 10.

### Statistics

Intervals between two contractions were determined for all recordings. The interval lengths of a single recording did not fulfil requirements of a normal distribution. Interval scatter was therefore defined as the length between 10^th^ and 90^th^ percentile (interdecile range, IDR). Statistical difference between groups of biological replicas were determined with Mann-Whitney U Test (*P<0.05).

## Supporting Information

Figure S1 **Cardiac differentiation.** Schematic illustration of EB formation and cardiomyocytes differentiation of hESC, adopted from Yang et al [2].
(TIF)

Figure S2 **Characterisation of non-myocyte content.** Quantitative PCR of markers for (myo)fibroblast (A: *ACTA2*, smooth muscle actin, B: *CNN1*, calponin), endothelial (C: *CDH5*, VE-cadherin, D: *PECAM1*, *CD31*, platelet/endothelial cell adhesion molecule), endodermal (E: *FOXA2*, forkhead box A2, F: *FOXA3*, forkhead box A3), mesodermal somite (G: *MEOX1*, mesenchyme homeobox 1) and neuroectodermal (H: *NEUROD1*, neurogenic differentiation 1, I: *SOX1* sex determining region Y-box 1) development of 2–3 week old EBs, 7–8 week old EBs and 5 week old EHTs, normalized to undifferentiated hESC, 4–6 biological replica. Bars show mean ± SD, *P<0.05 (Student's *t*-test), bars show means ± SD.
(TIF)

Figure S3 **Electrophysiological characterisation.** Spontaneous electrical activity recorded in hESC-CMs in the current clamp mode. Representative recordings of APs under different culture conditions: A EB (2–3 weeks), B EBs (7–8 weeks), C EHT (*left panel*) shown on an expanded time scale (*middle panel*) and exemplary effects of Tetrodotoxin (TTX) and E-4031 on AP morphology (*right panel*). For EHTs, examples of APs of relatively short (with associated nisoldipine effect) and long duration are shown.
(TIF)

Movie S1 **Beating embryoid bodies.** Spontaneous contractions of human embryoid bodies, 10× magnification.
(AVI)

Movie S2 **Beating human EHT.** Spontaneous and regular contractions of human EHT spanned between two silicone posts.
(AVI)

Table S1 **PCR primers.** List of primer pairs used to determine transcript levels by quantitative PCR.
(XLS)

Information S1 **Full set of original hEHT contractility recordings under baseline conditions and in the presence proarrhythmic compounds, analysis of parameters of contractility.**
(PDF)
