# Supplementary figures and images for: Human Engineered Heart Tissue as a Versatile Tool in Basic Research and Preclinical Toxicology
Source: PLoS One. 2011 Oct 20;6(10):e26397. doi: 10.1371/journal.pone.0026397 (PMC3197640; doi:10.1371/journal.pone.0026397)

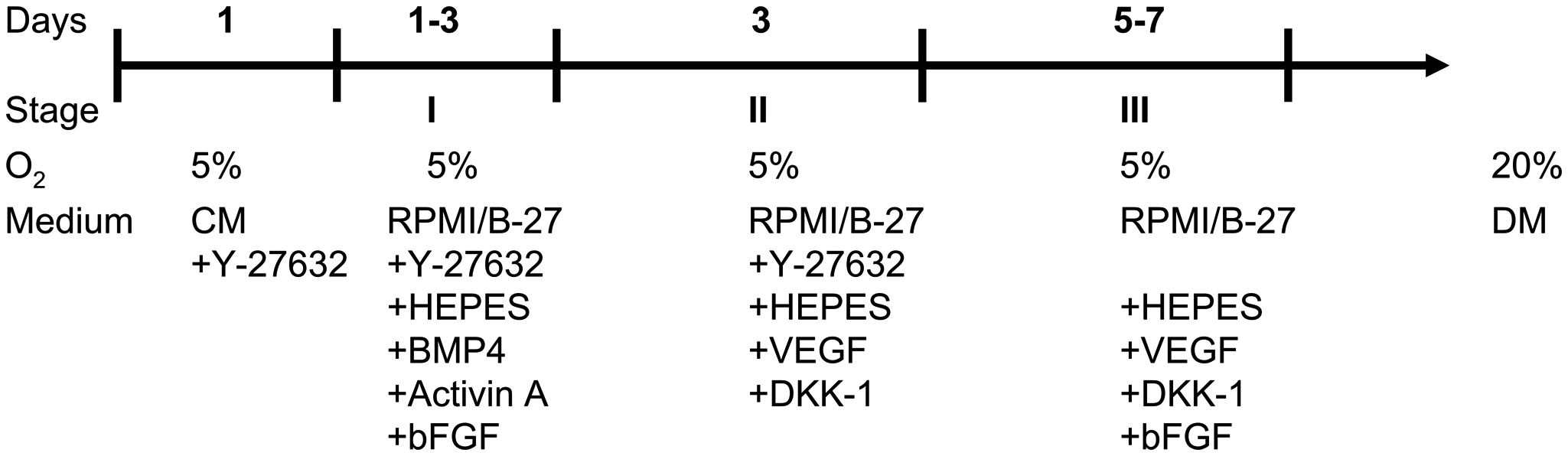

Supplement: Figure S1 — Cardiac differentiation. Schematic illustration of EB formation and cardiomyocytes differentiation of hESC, adopted from Yang et al [2]. (TIF) [file pone.0026397.s001.tif]

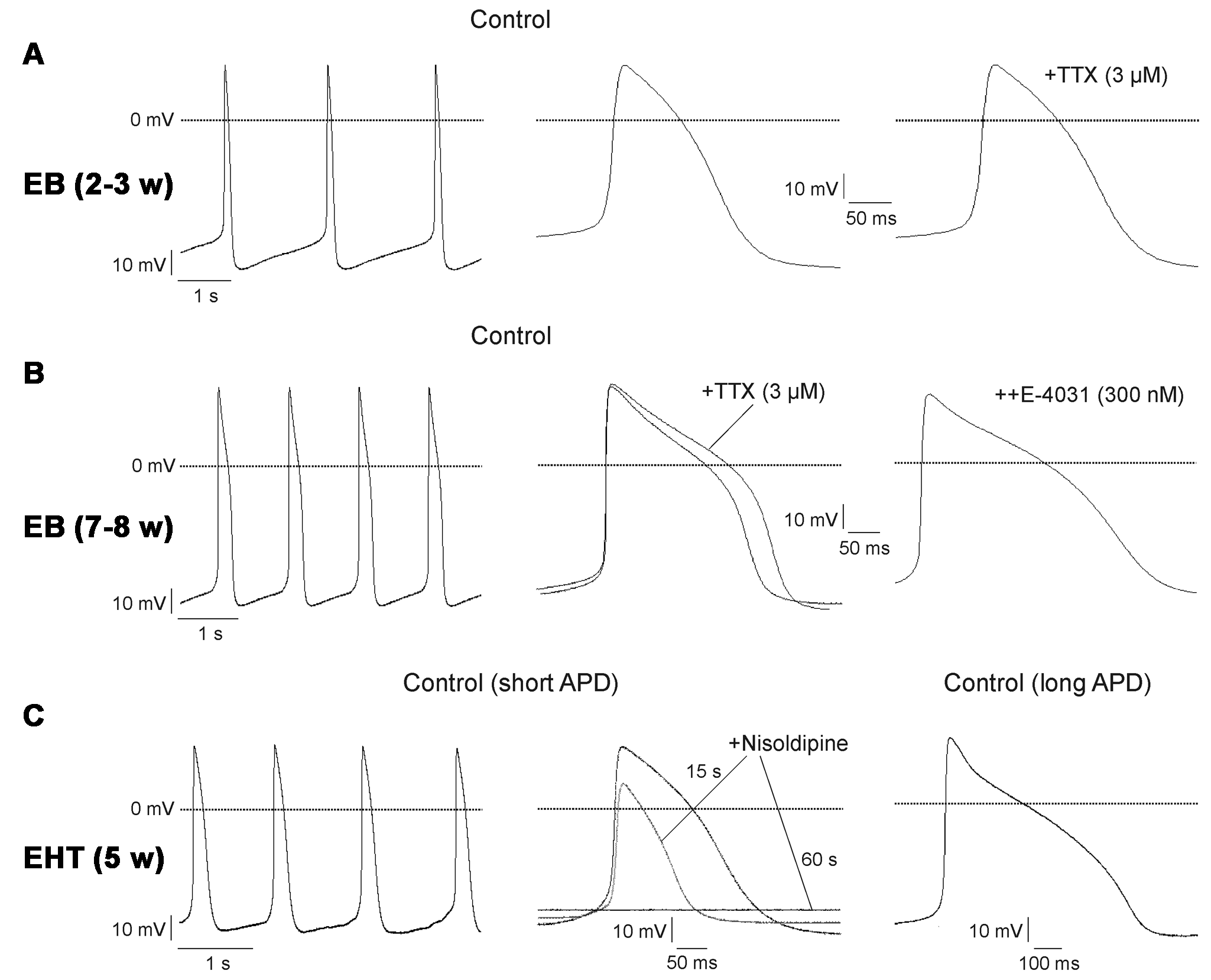

Supplement: Figure S2 — Characterisation of non-myocyte content. Quantitative PCR of markers for (myo)fibroblast (A: ACTA2, smooth muscle actin, B: CNN1, calponin), endothelial (C: CDH5, VE-cadherin, D: PECAM1, CD31, platelet/endothelial cell adhesion molecule), endodermal (E: FOXA2, forkhead box A2, F: FOXA3, forkhead box A3), mesodermal somite (G: MEOX1, mesenchyme homeobox 1) and neuroectodermal (H: NEUROD1, neurogenic differentiation 1, I: SOX1 sex determining region Y-box 1) development of 2–3 week old EBs, 7–8 week old EBs and 5 week old EHTs, normalized to undifferentiated hESC, 4–6 biological replica. Bars show mean ± SD, *P<0.05 (Student's t-test), bars show means ± SD. (TIF) [file pone.0026397.s002.tif]

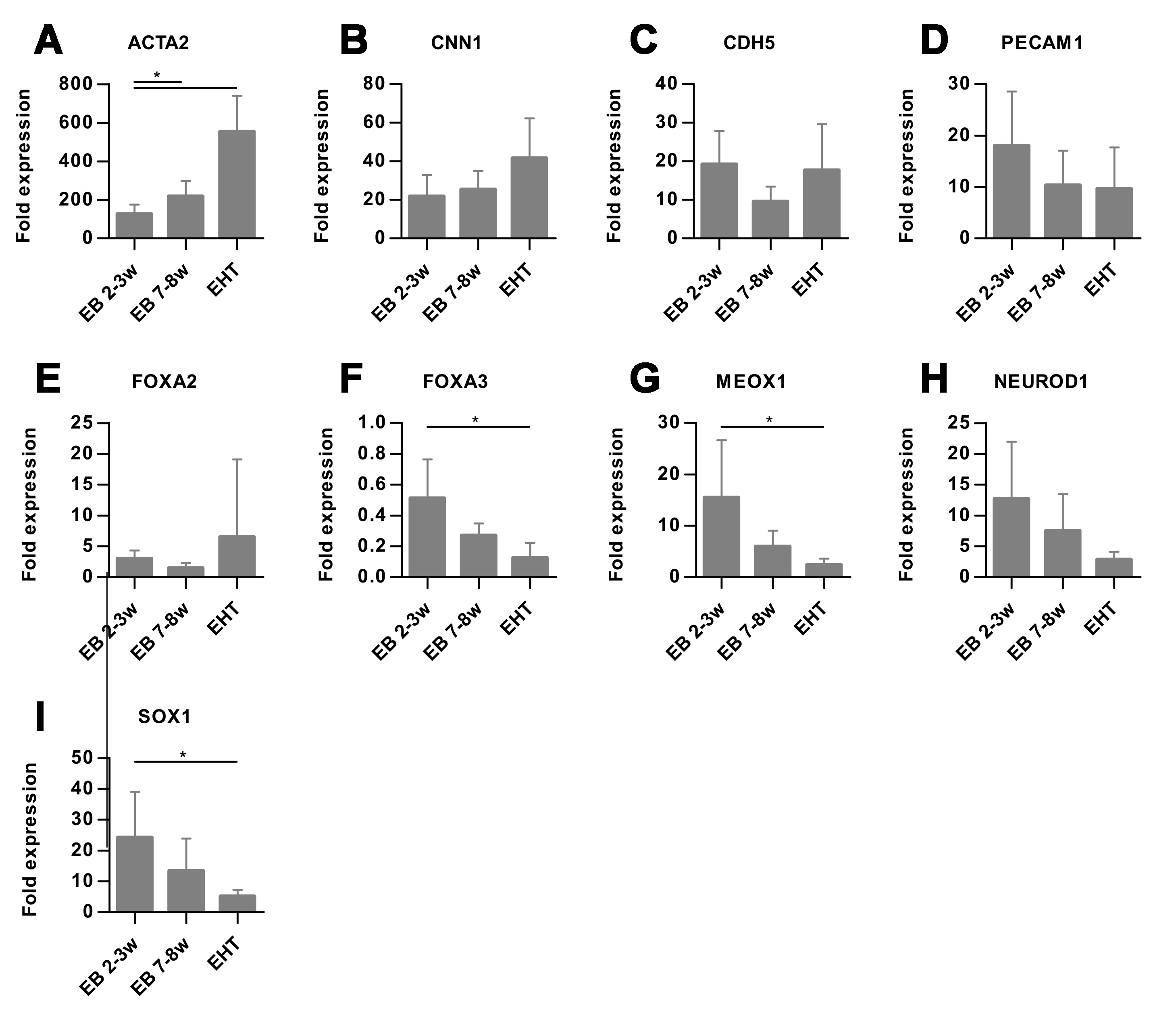

Supplement: Figure S3 — Electrophysiological characterisation. Spontaneous electrical activity recorded in hESC-CMs in the current clamp mode. Representative recordings of APs under different culture conditions: A EB (2–3 weeks), B EBs (7–8 weeks), C EHT (left panel) shown on an expanded time scale (middle panel) and exemplary effects of Tetrodotoxin (TTX) and E-4031 on AP morphology (right panel). For EHTs, examples of APs of relatively short (with associated nisoldipine effect) and long duration are shown. (TIF) [file pone.0026397.s003.tif]
