# Supplementary material for: Human Engineered Heart Tissue as a Versatile Tool in Basic Research and Preclinical Toxicology
Source: PLoS One. 2011 Oct 20;6(10):e26397. doi: 10.1371/journal.pone.0026397 (PMC3197640; doi:10.1371/journal.pone.0026397)
Supplement: Information S1 — Full set of original hEHT contractility recordings under baseline conditions and in the presence proarrhythmic compounds, analysis of parameters of contractility. (PDF) [file pone.0026397.s007.pdf]

Supporting Information S1

Original recordings and analysis

Original recordings of contraction patterns of four EHTs are presented under baseline condition and in the presence of proarrhythmic compounds.

E-4031 original recordings

E-4031 baseline

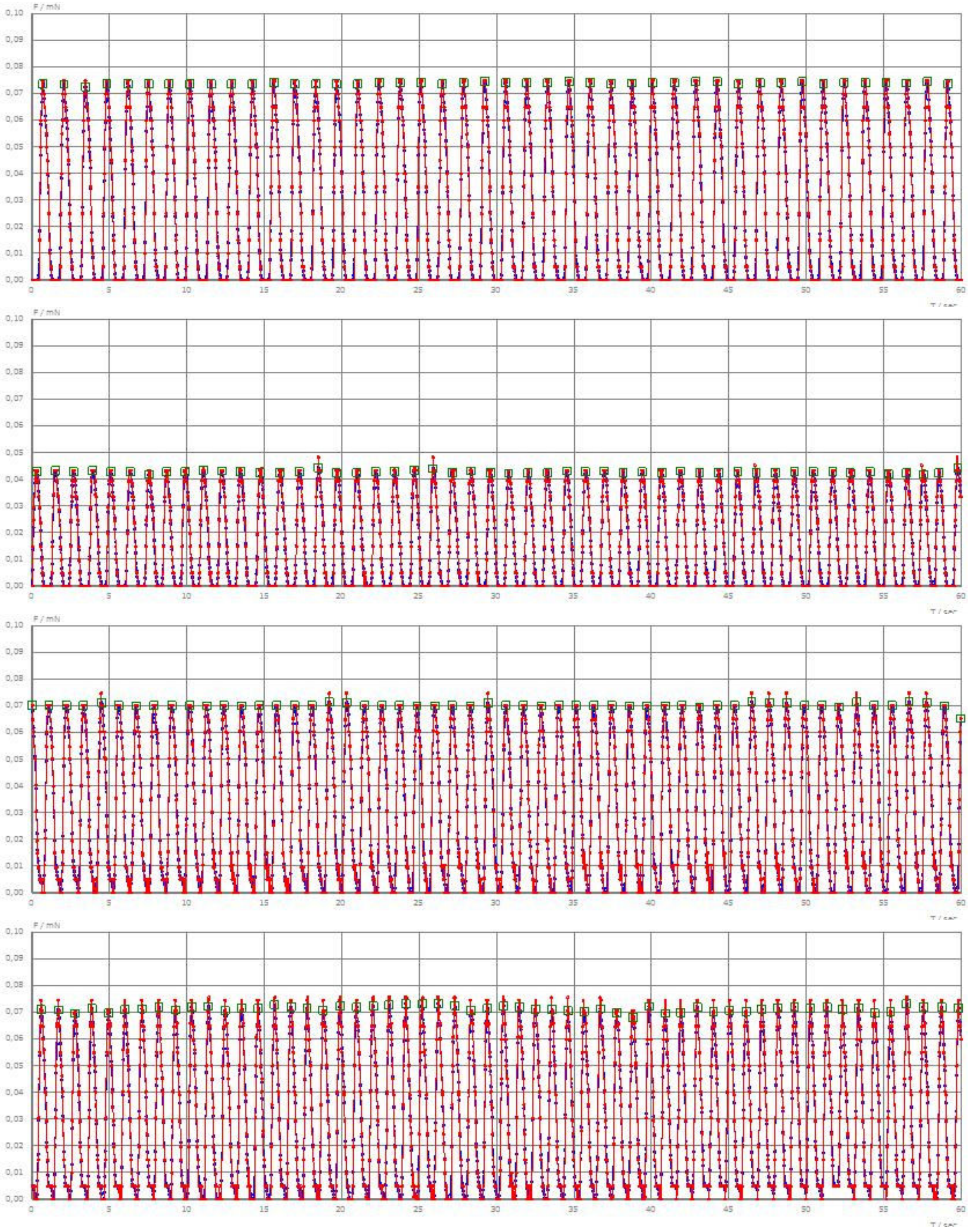

E-4031 1 nM

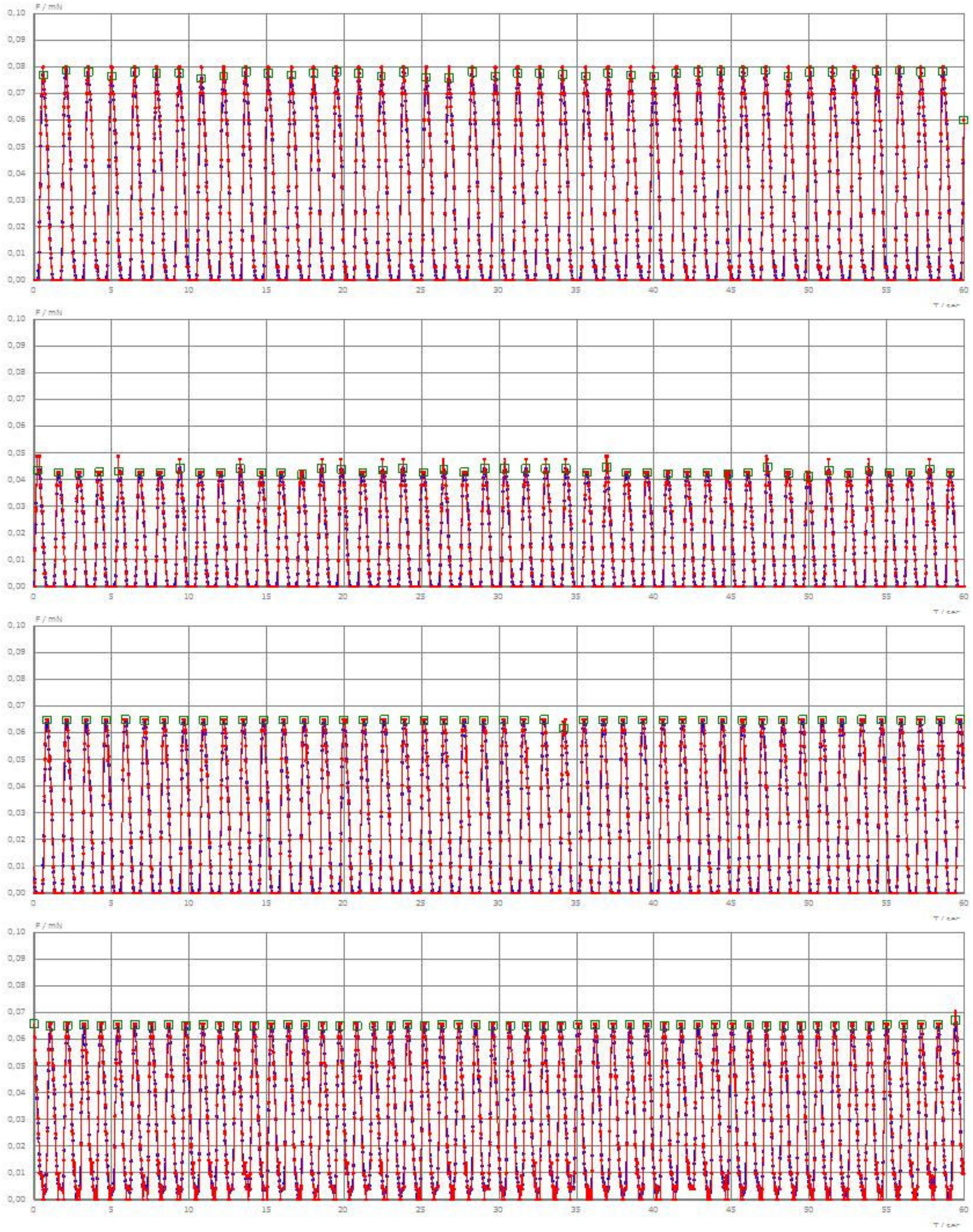
F / mN
0,10
0,09
0,08
0,07
0,06
0,05
0,04
0,03
0,02
0,01
0,00
0
5
10
15
20
25
30
35
40
45
50
55
60

E-4031 3 nM

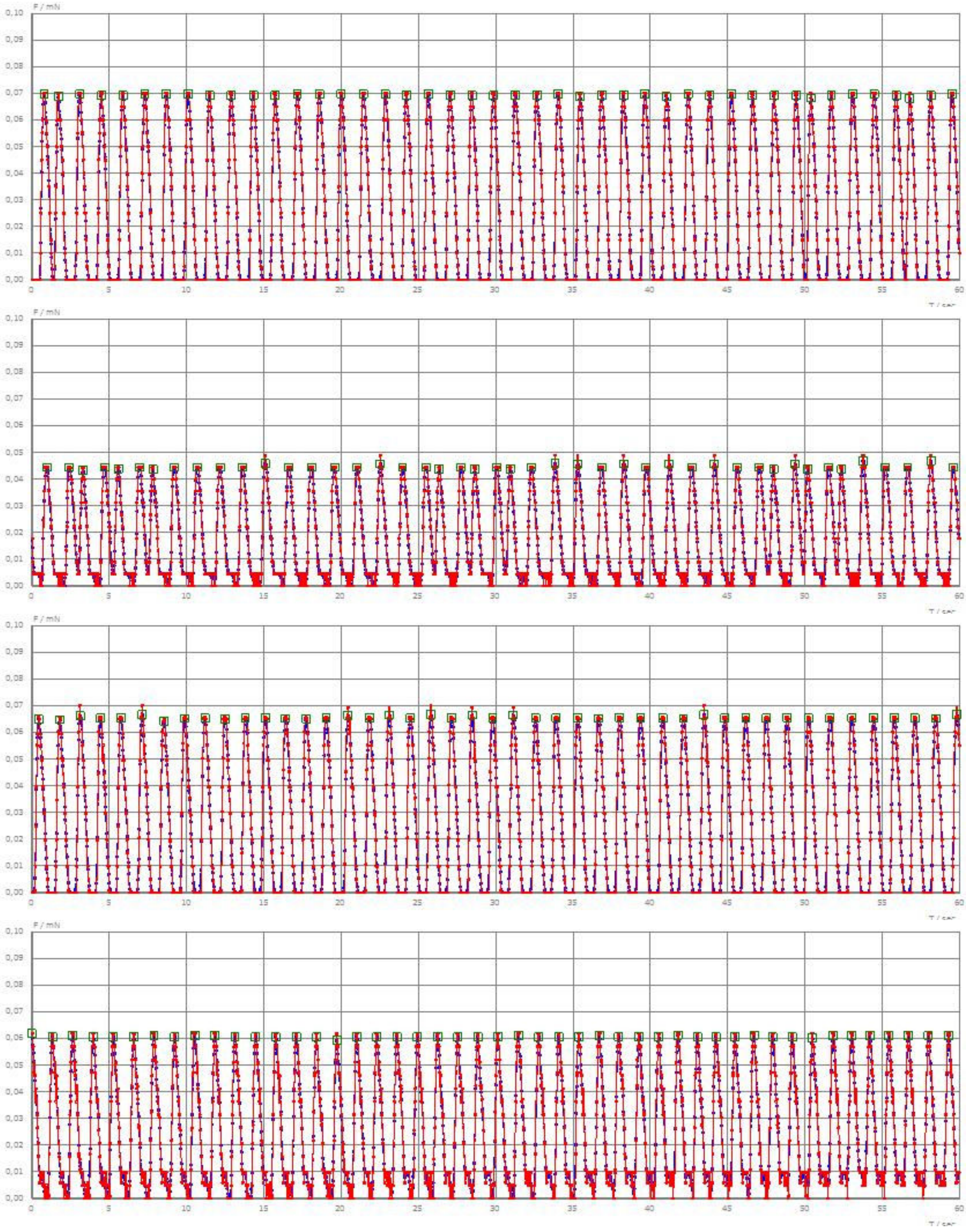

E-4031 10 nM

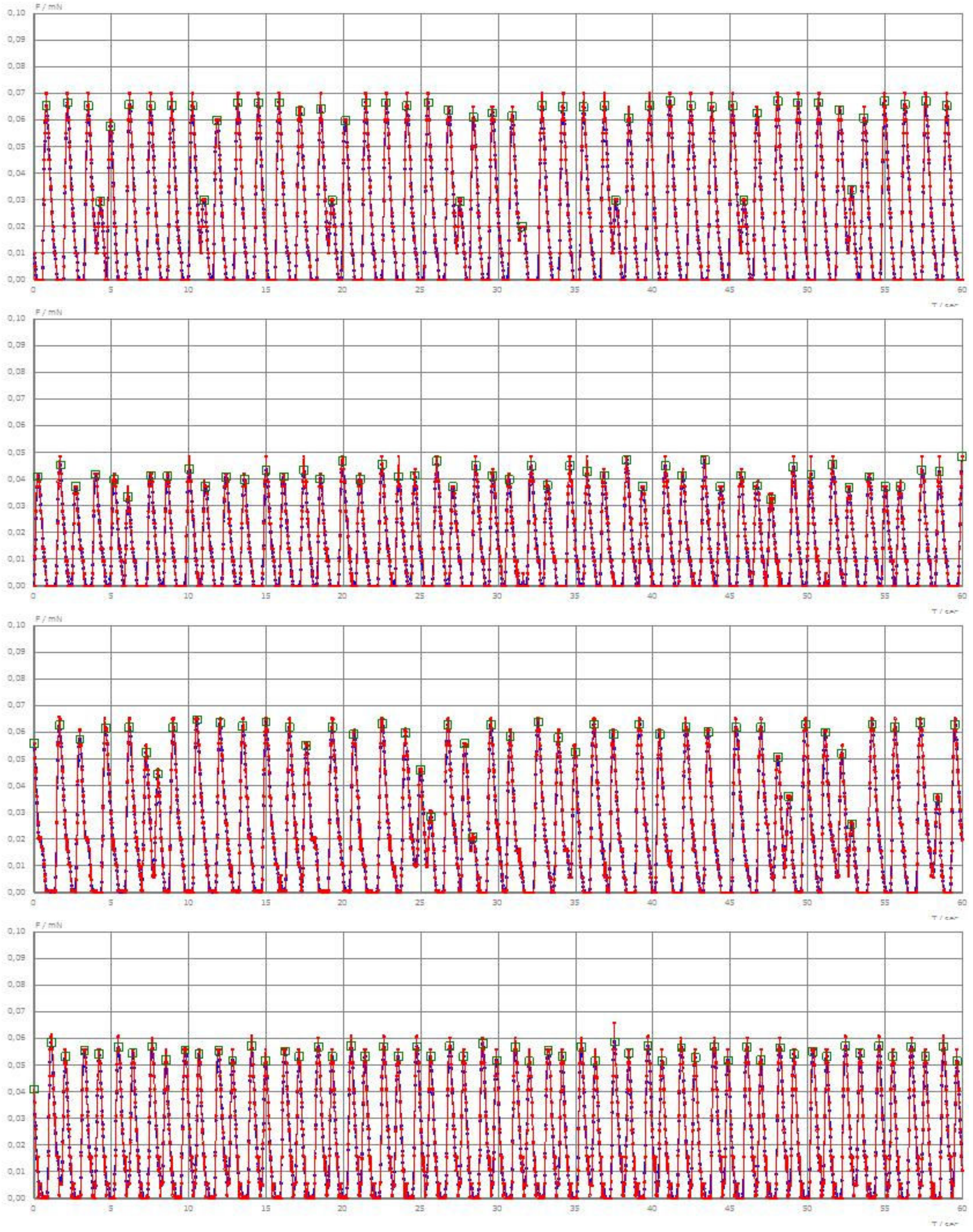

E-4031 30 nM

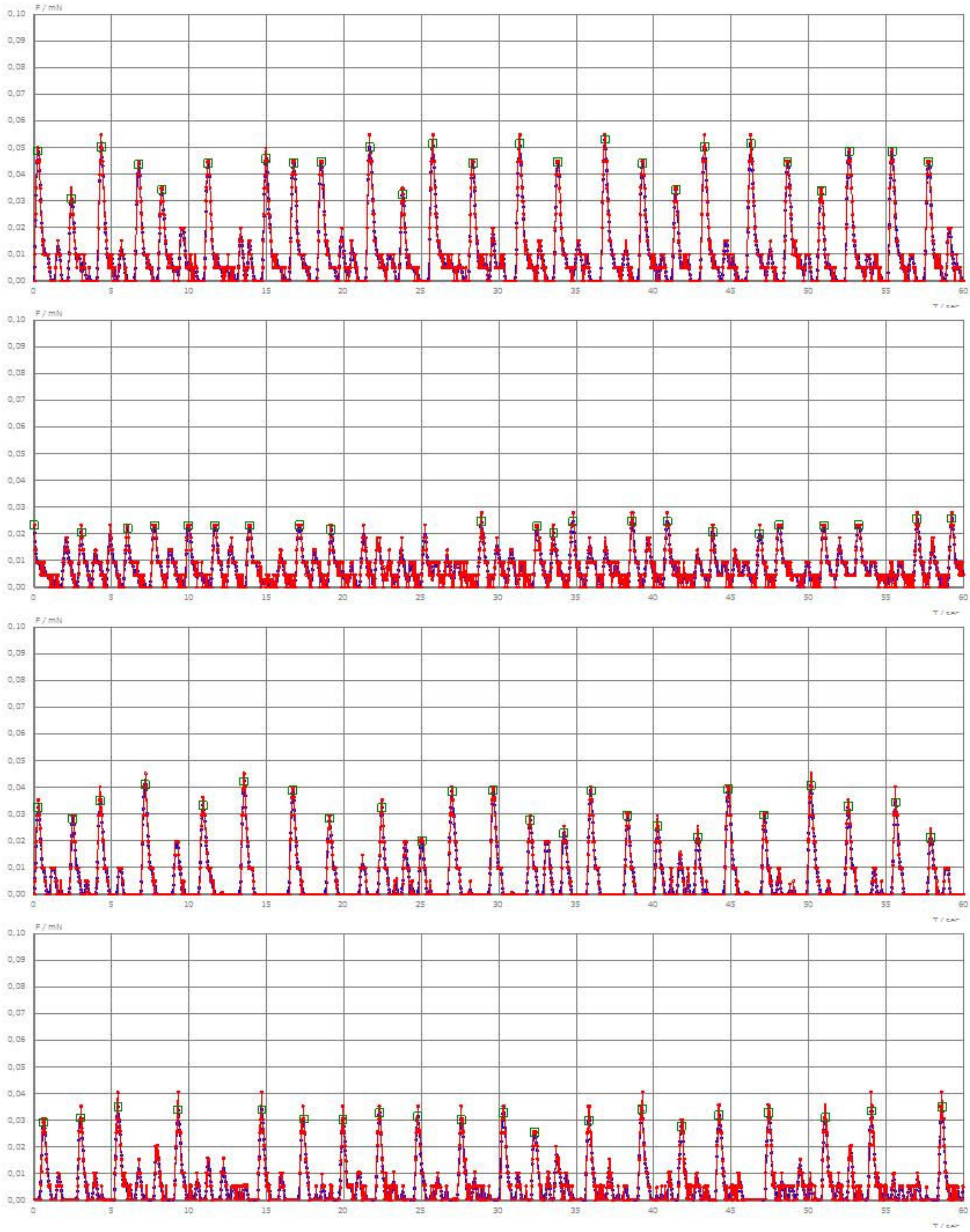

Analysis E-4031

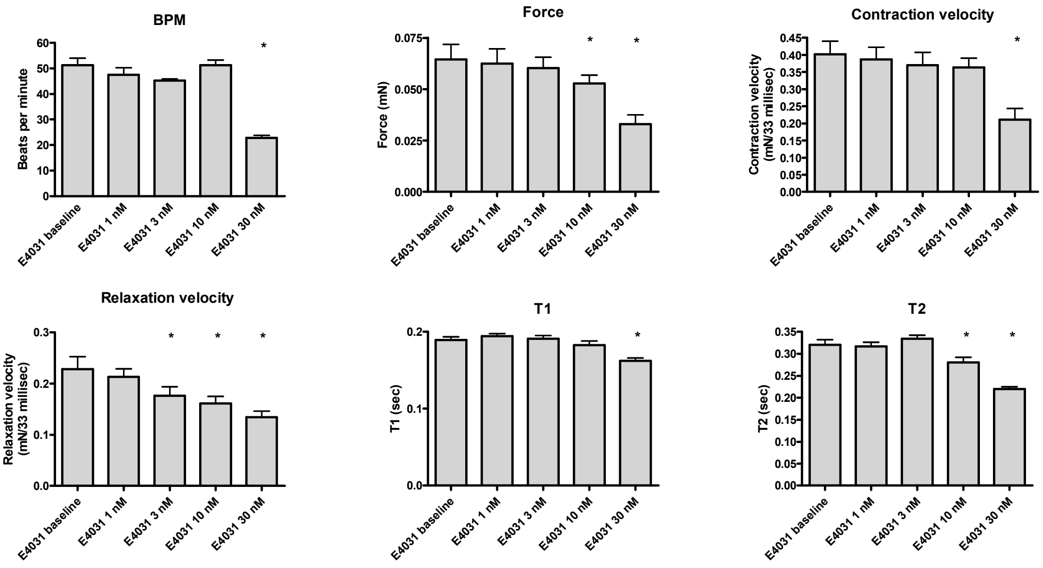

Analysis of contraction. **P*<0.05 (Student's t-test), 4 biological replicas, bars show means ± SD. Beats per minute (BPM), contraction time (T1), relaxation time (T2).

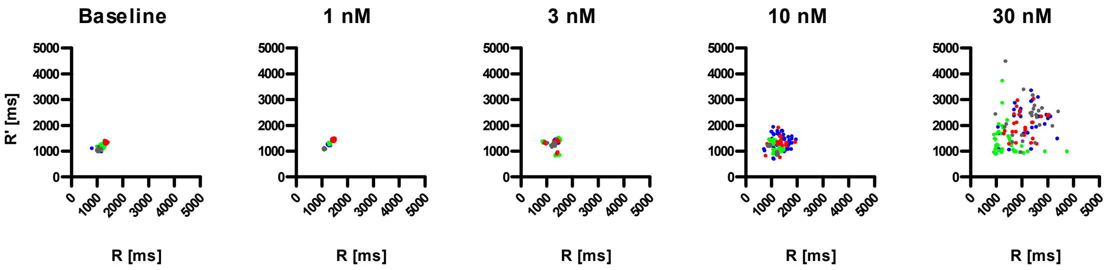

Graphical illustration of irregularity integrating 4 biological replicas. Ordinates indicate the distance from a given twitch to the following, the abscissa the distance to the previous twitch. Biological replicas are discriminated by color code.

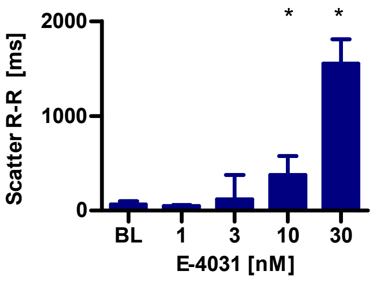

Scatter of beat-to-beat variability in the presence of E-4031, **P*<0.05 (Mann-Whitney U test), 4 biological replicas, bars show median ± interquartile range.

.

Procainamide - original recordings

Procainamide baseline

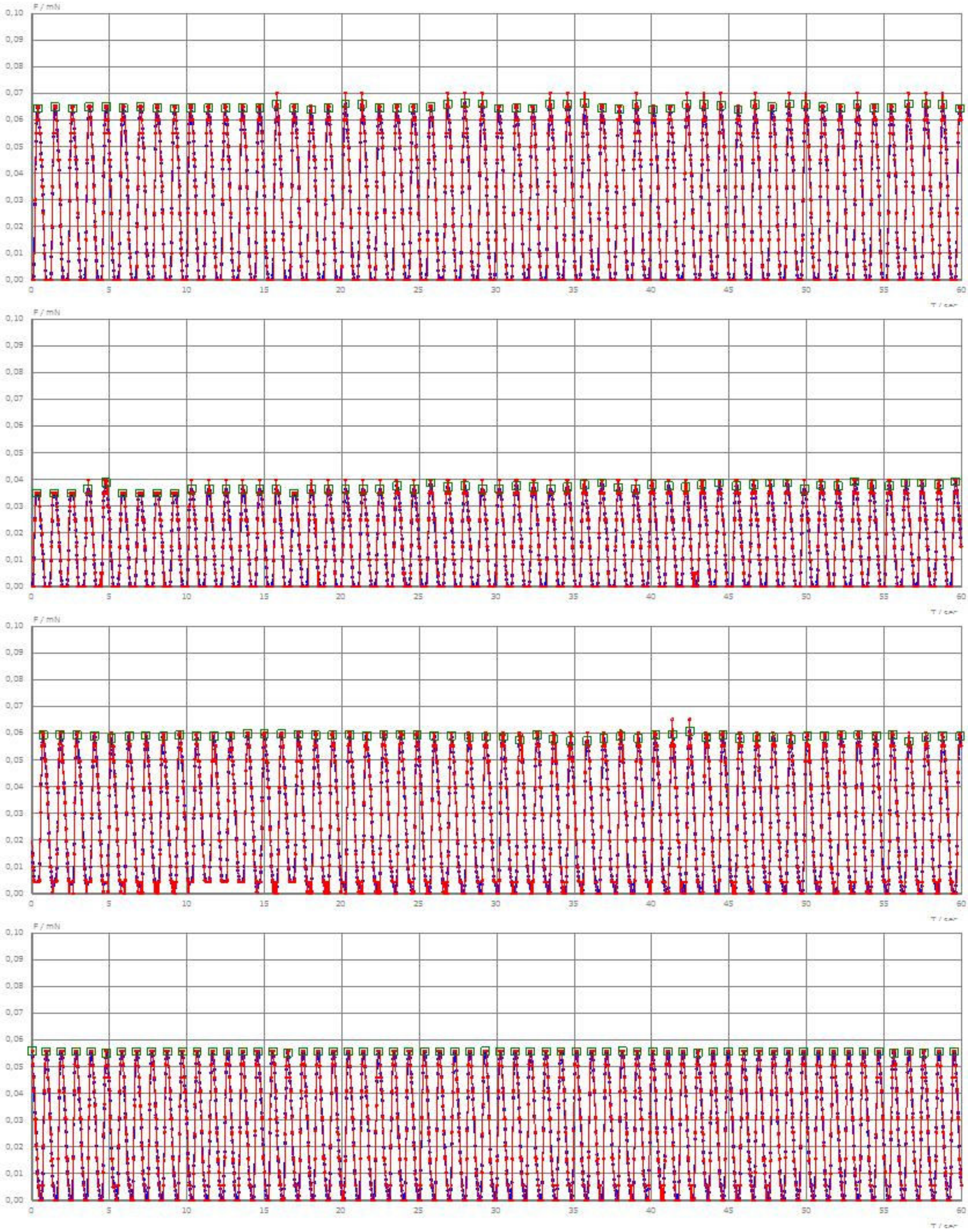

Procainamide 1 μM

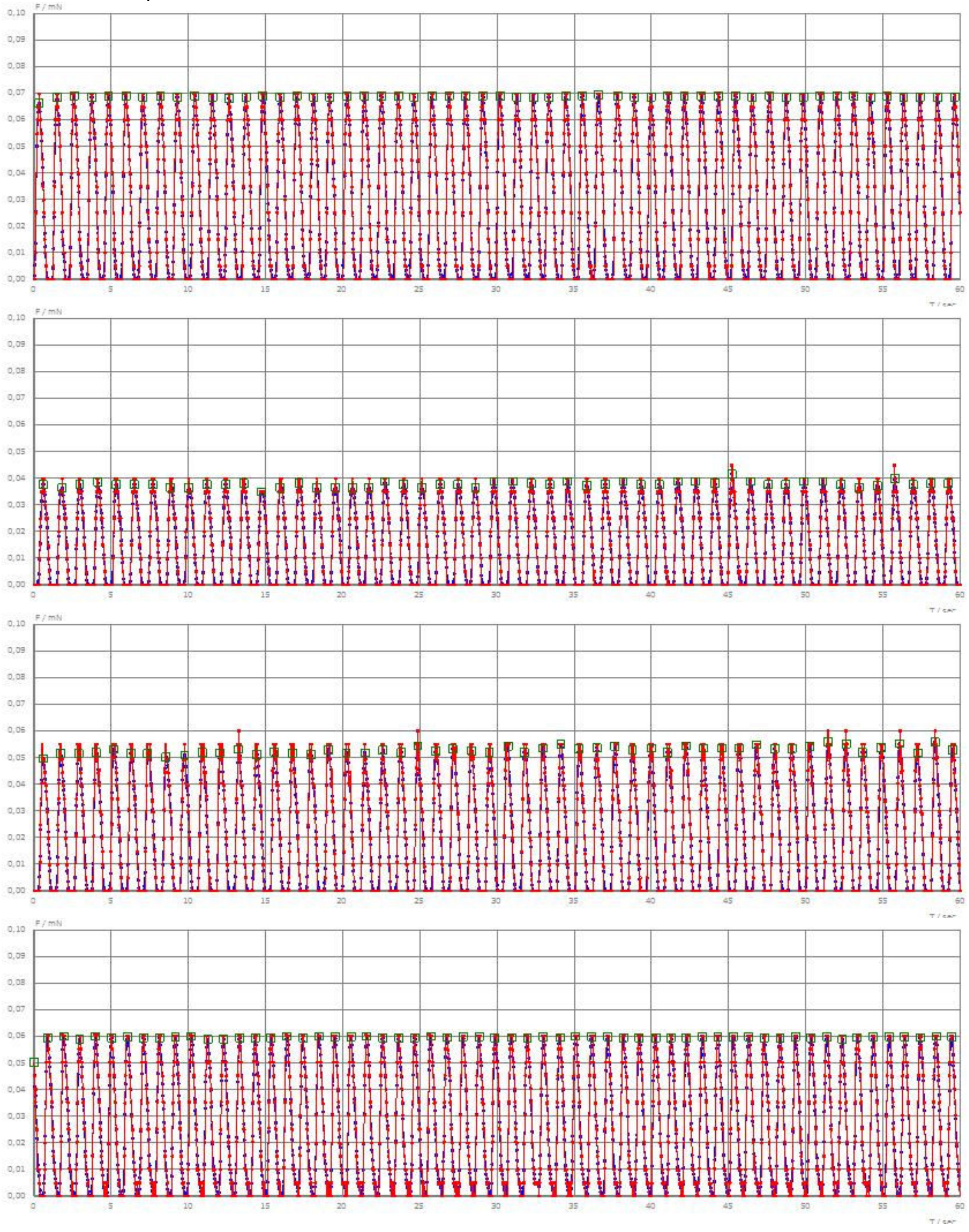

Procainamide 10 µM

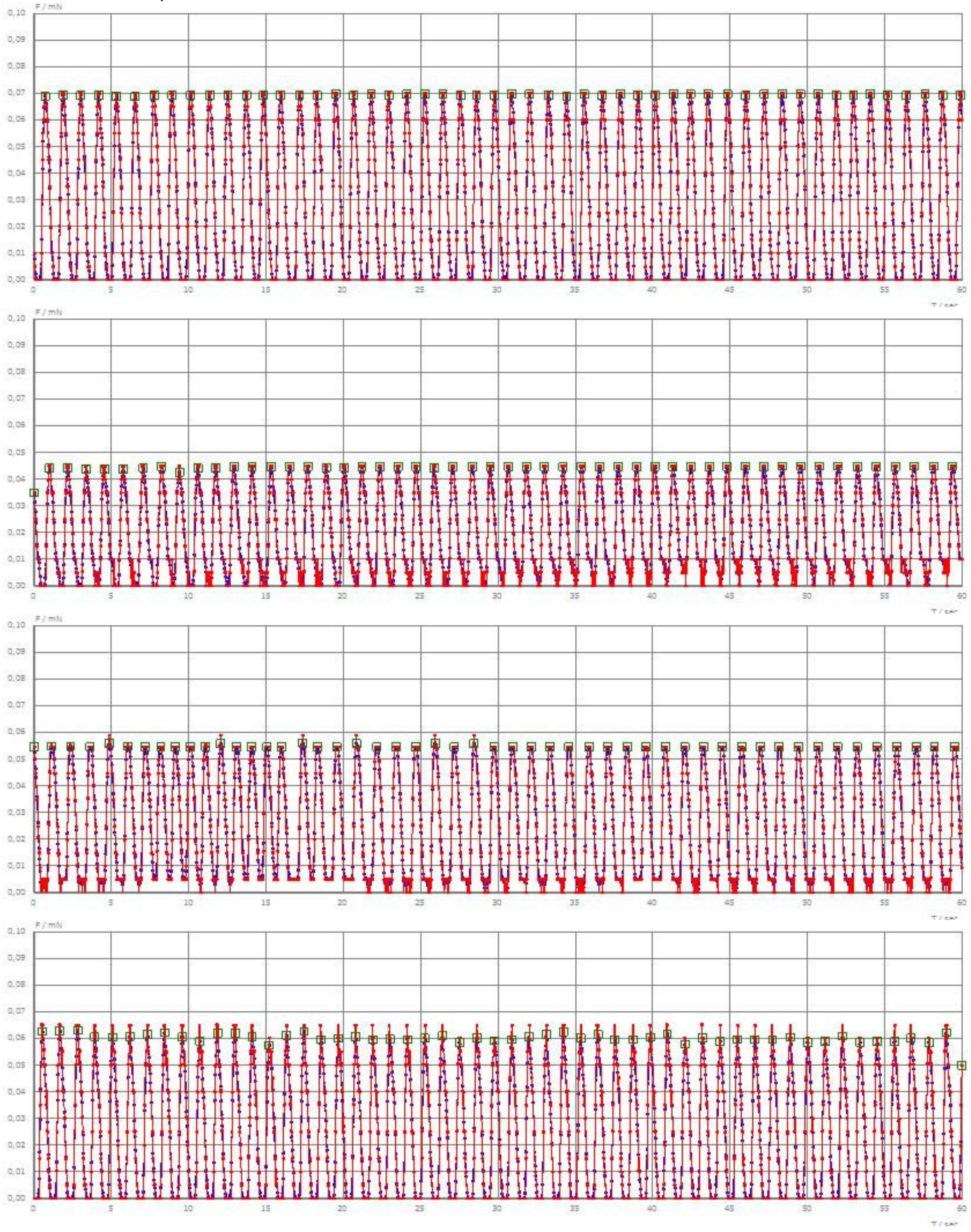

Procainamide 30 µM

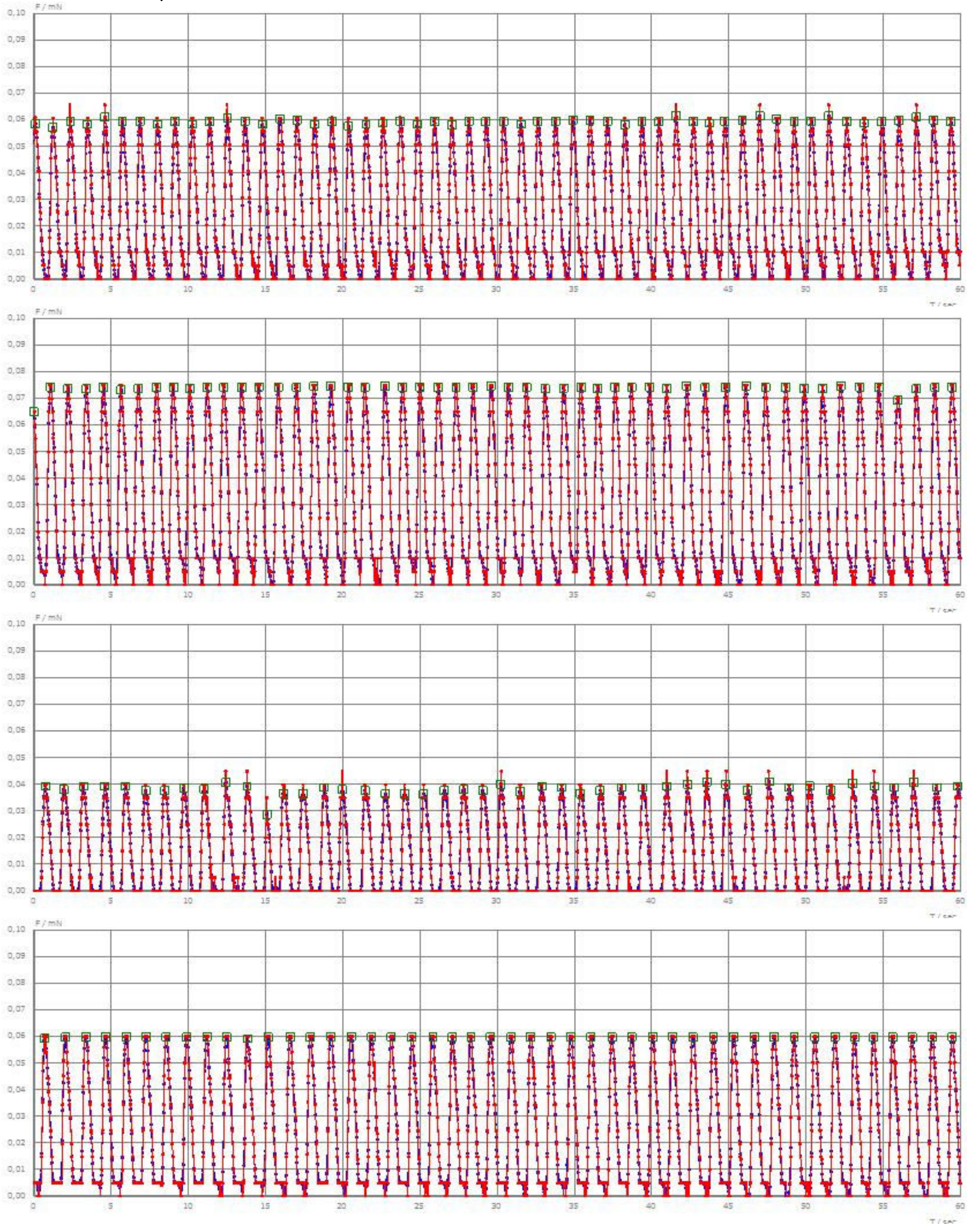

Procainamide 100 µM

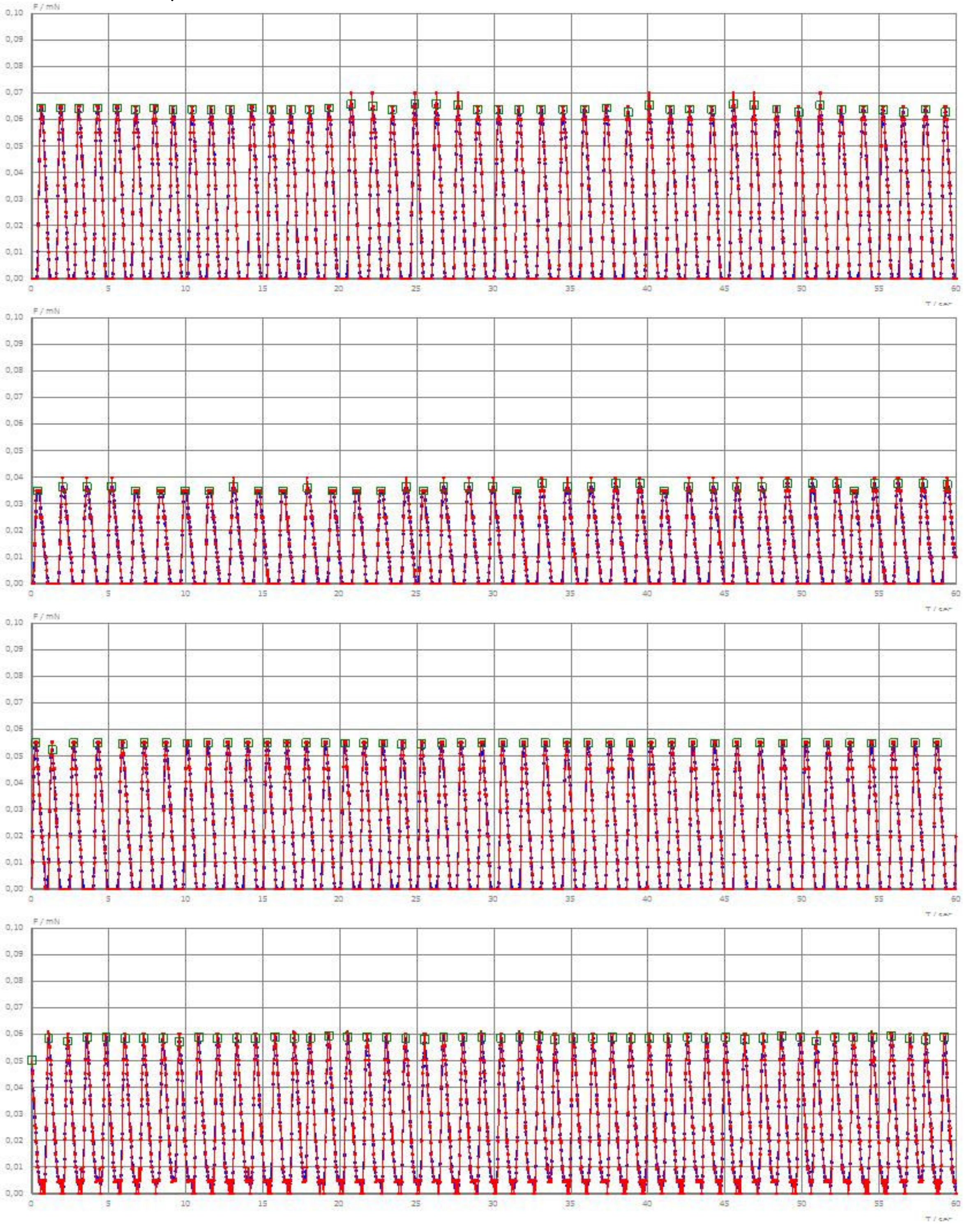

Procainamide 300 μM

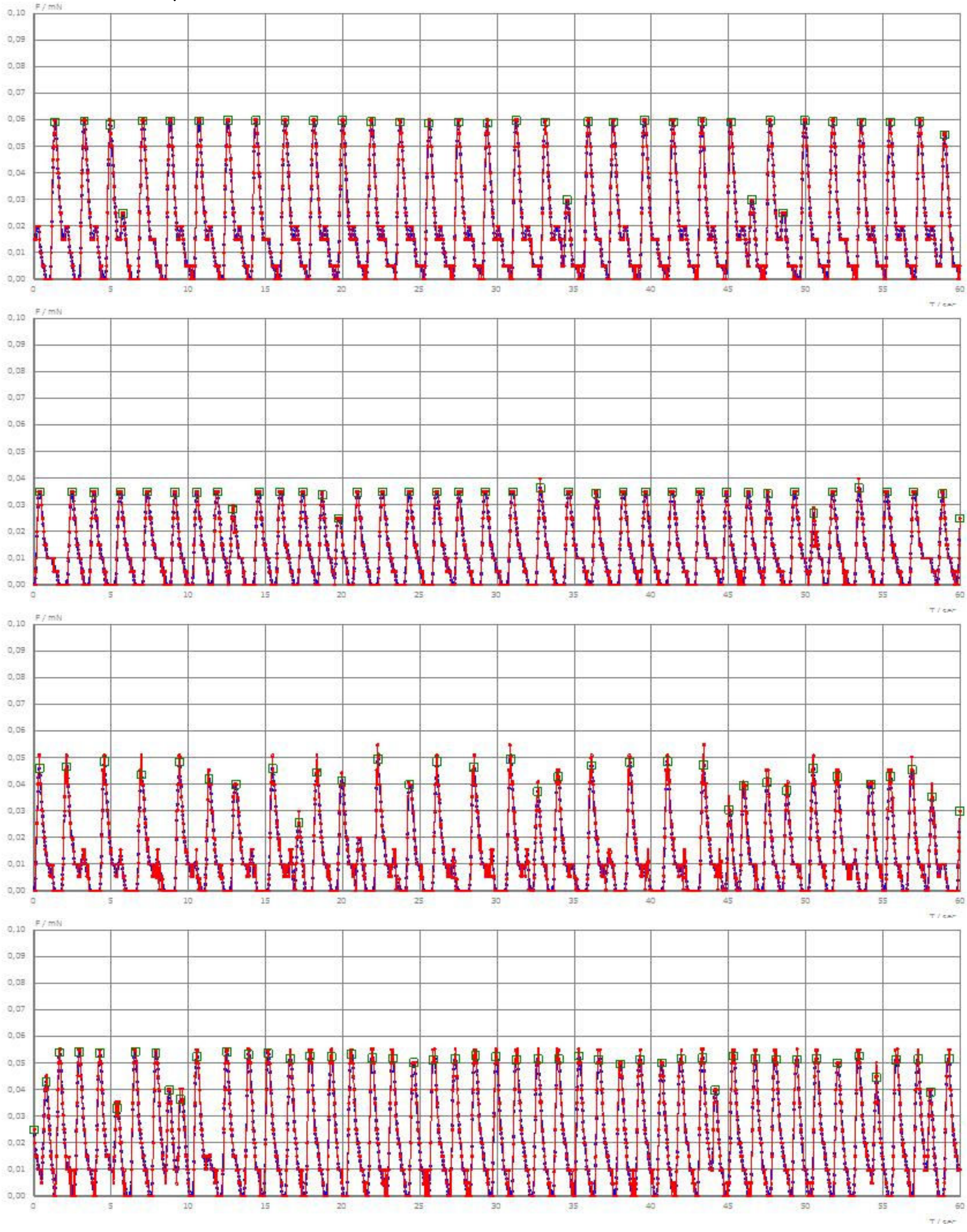

Analysis procainamide

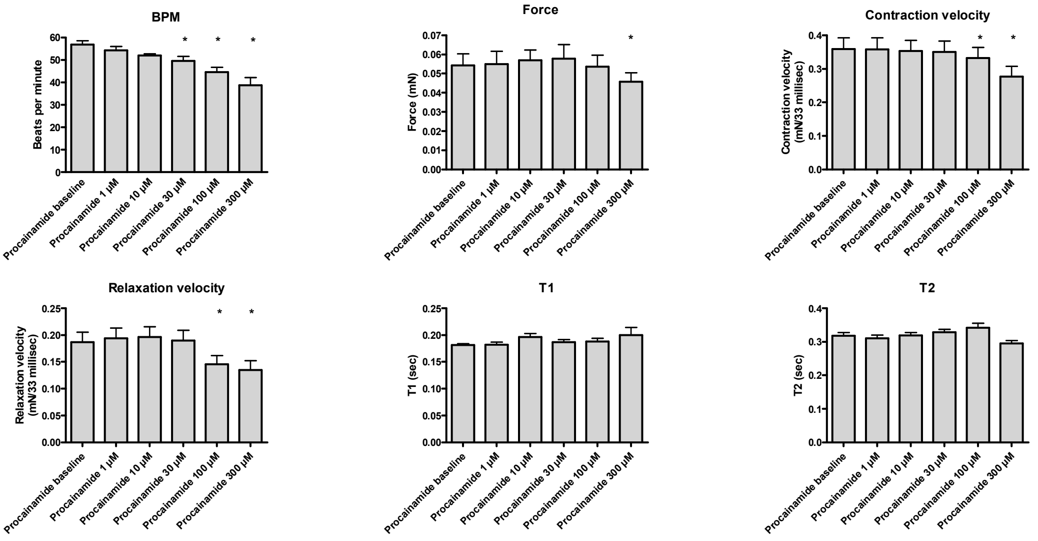

Analysis of contraction. **P*<0.05 **(**Student's t-test**),** 4 biological replicas, bars show means ± SD. Beats per minute (BPM), contraction time (T1), relaxation time (T2).

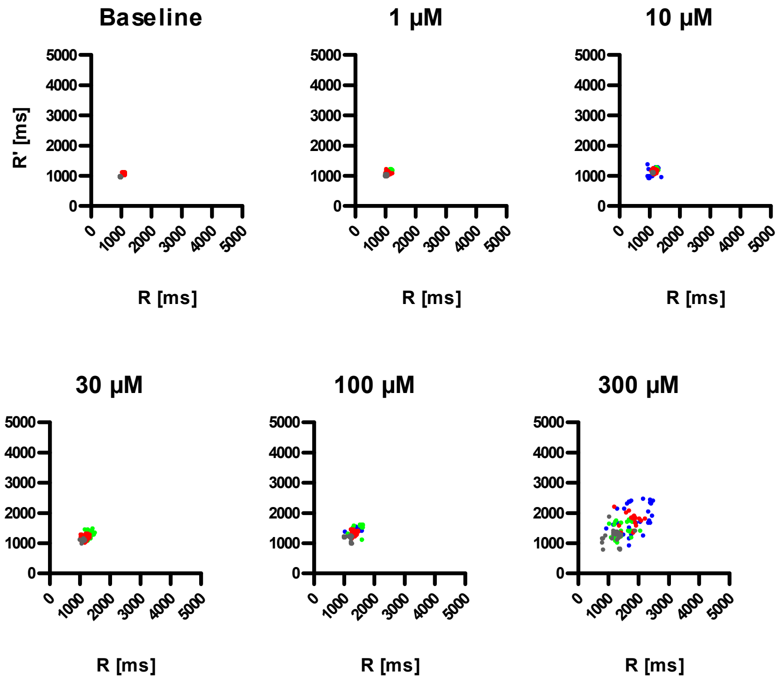

Graphical illustration of irregularity integrating 4 biological replicas. Ordinates indicate the distance from a given twitch to the following, the abscissa the distance to the previous twitch. Biological replicas are discriminated by color code.

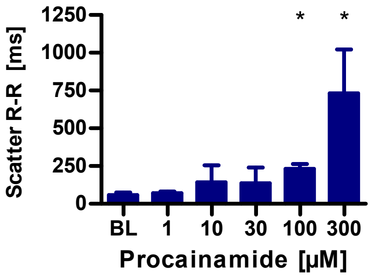

Scatter of beat-to-beat variability in the presence of procainamide, *P<0.05 (Mann-Whitney U test), 4 biological replicas, bars show median ± interquartile range.

Quinidine - original recordings

Quinidine baseline

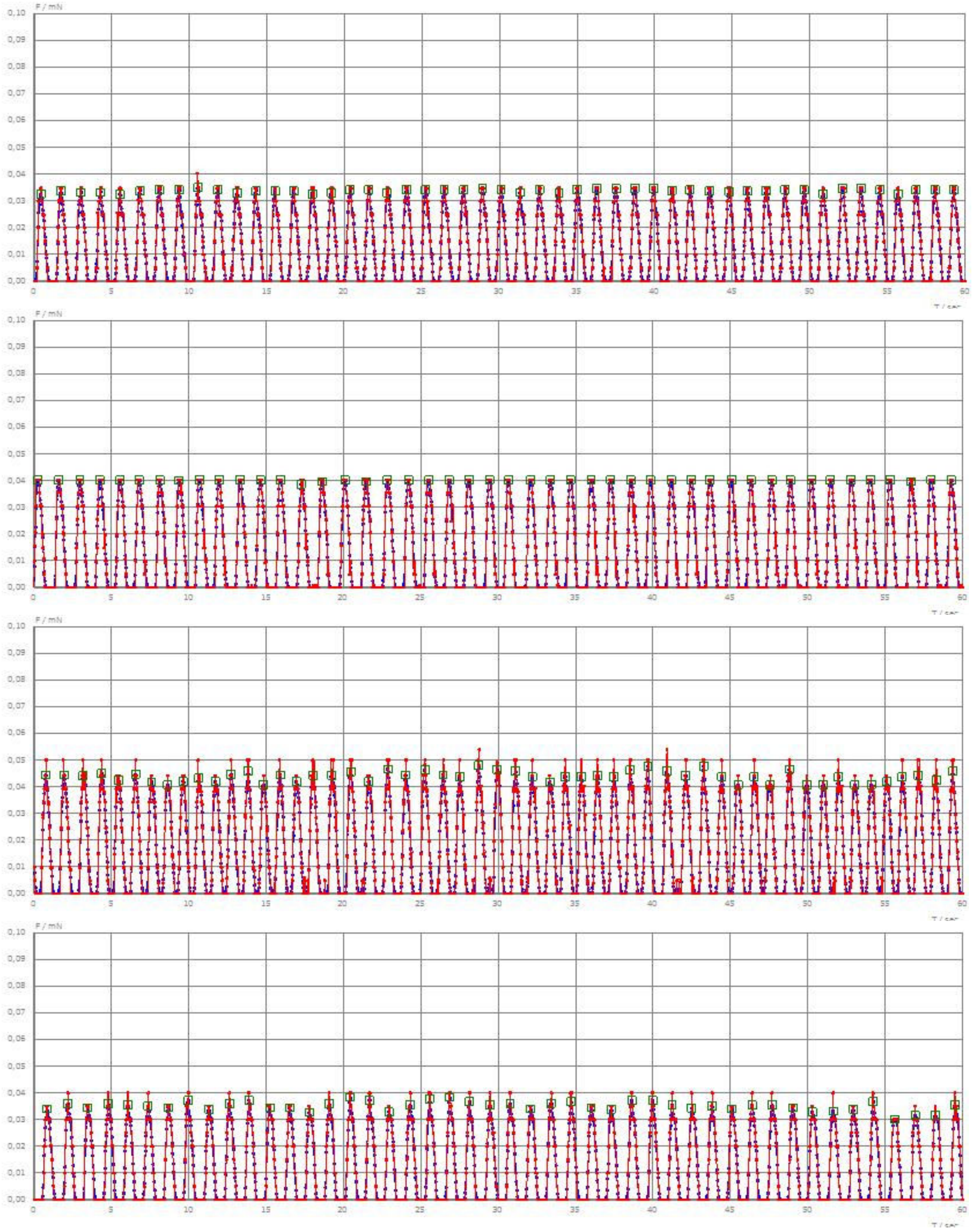

Quinidine 10 nM

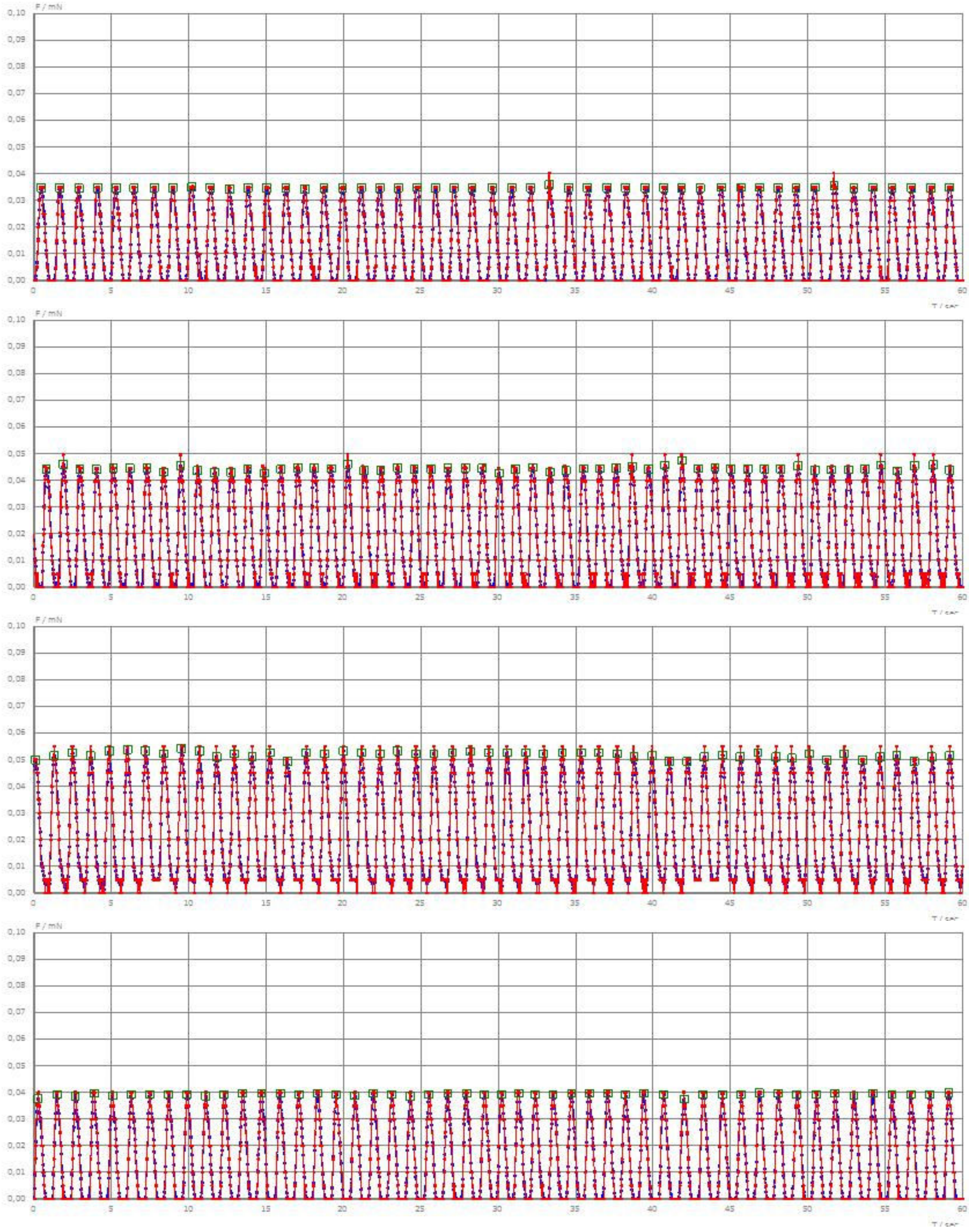

Quinidine 100 nM

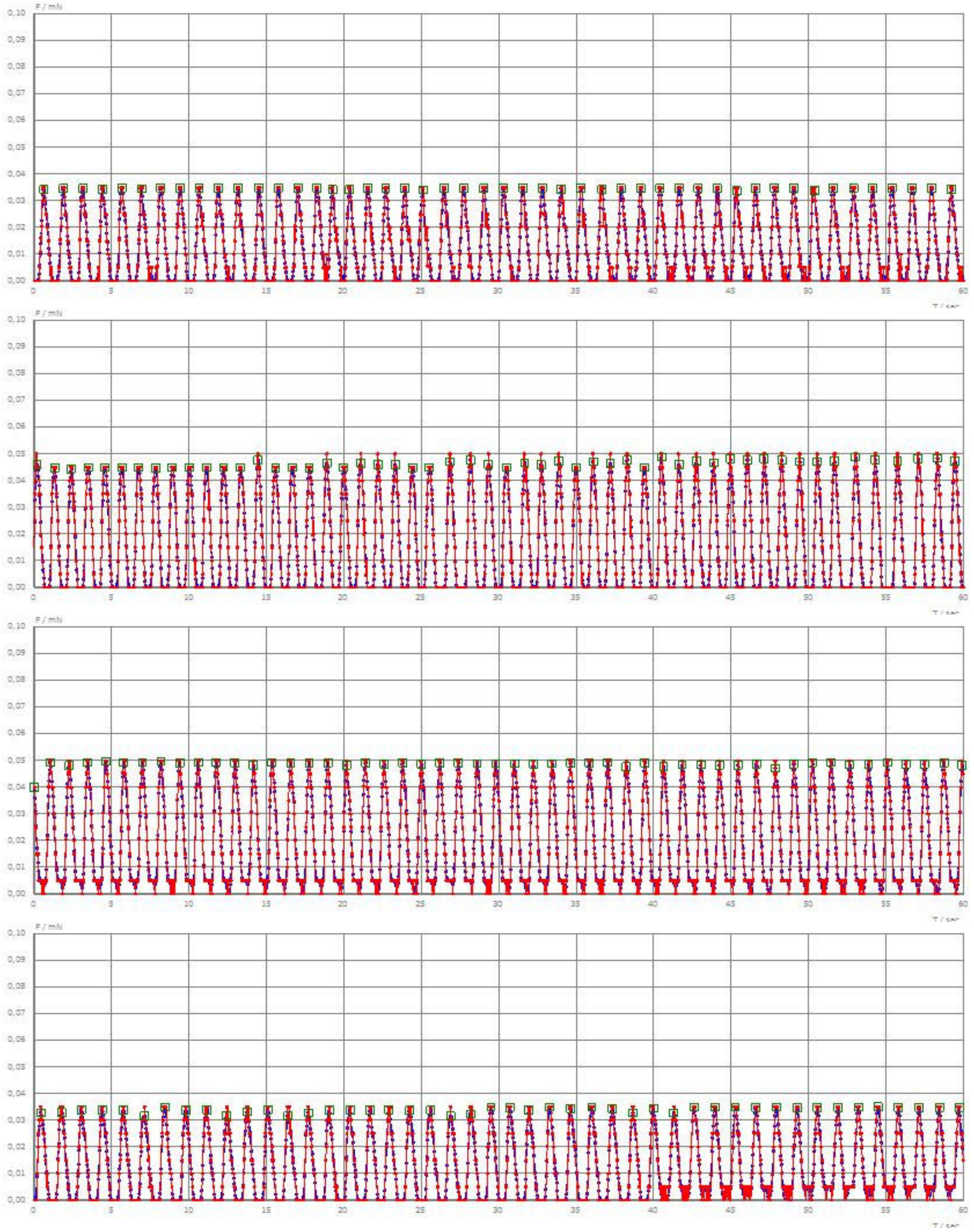

Quinidine 300 nM

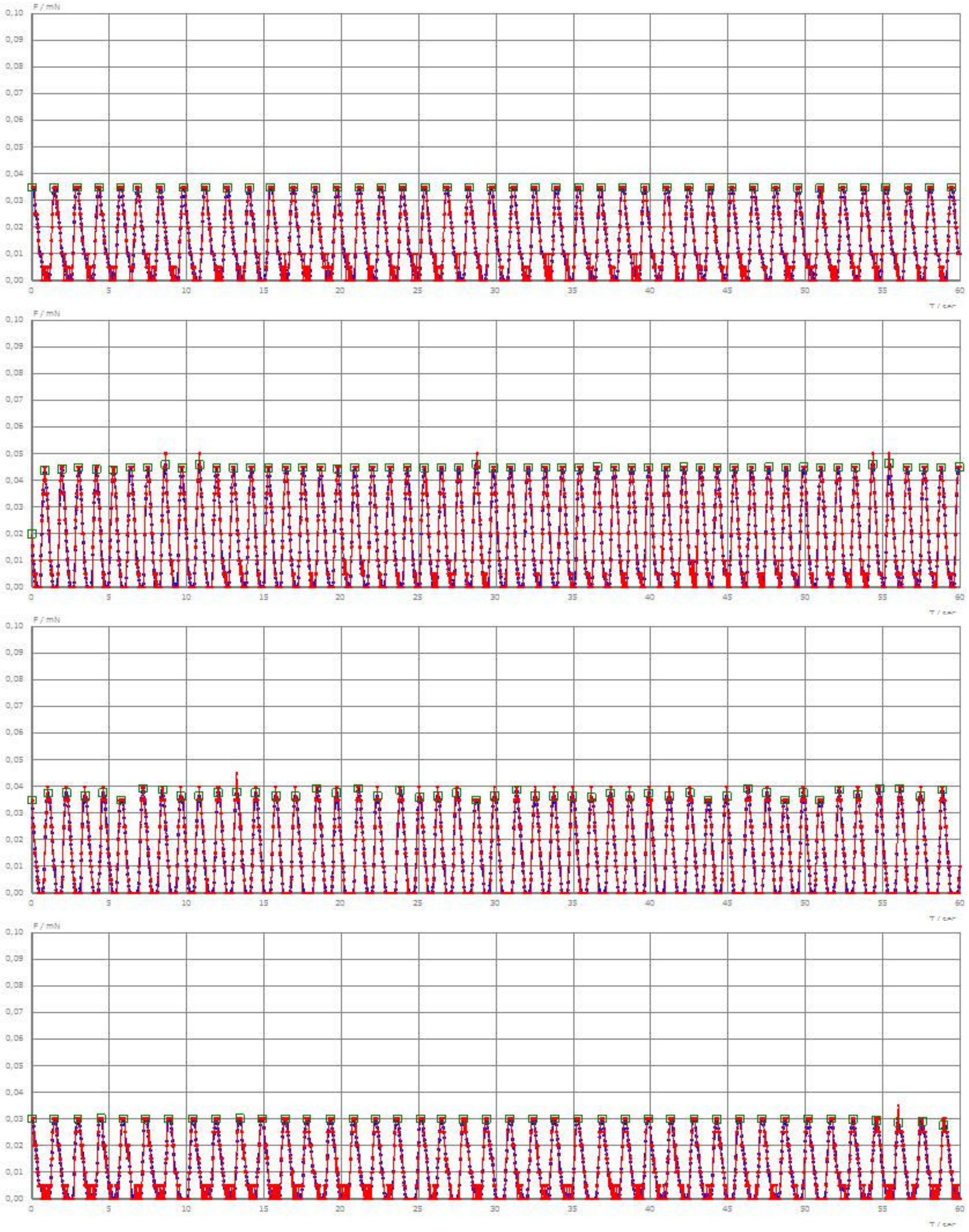

Quinidine 1000 nM

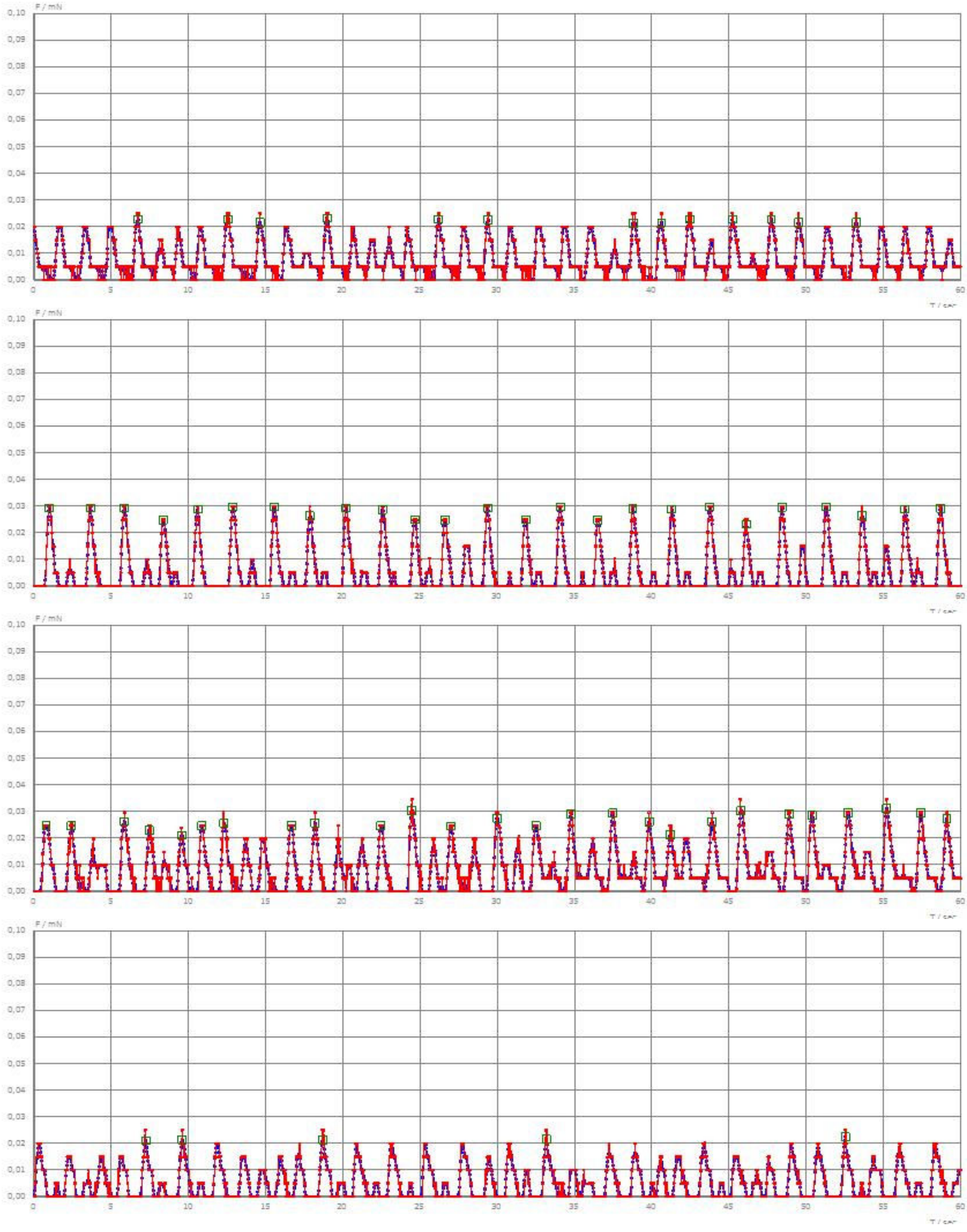

Analysis quinidine

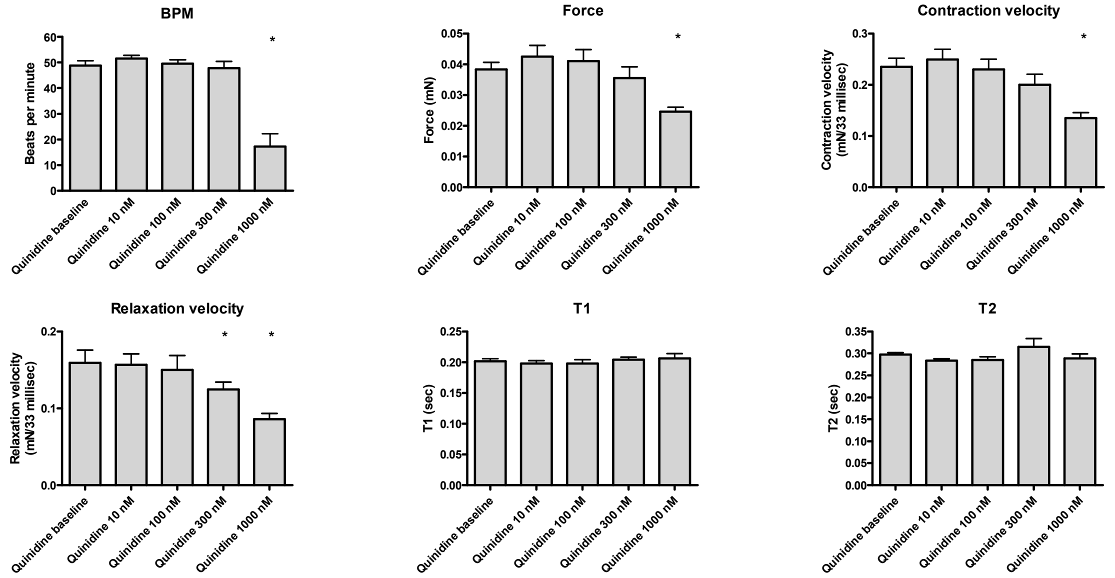

Analysis of contraction. *$P<0.05$ (Student's t-test), 4 biological replicas, bars show means ± SD. Beats per minute (BPM), contraction time (T1), relaxation time (T2).

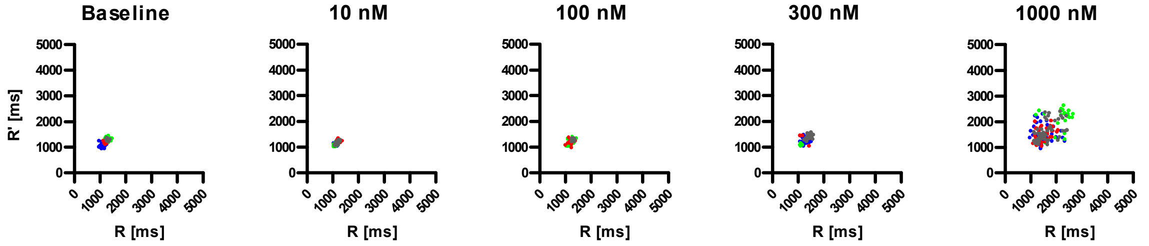

Graphical illustration of irregularity integrating 4 biological replicas. Ordinates indicate the distance from a given twitch to the following, the abscissa the distance to the previous twitch. Biological replicas are discriminated by color code.

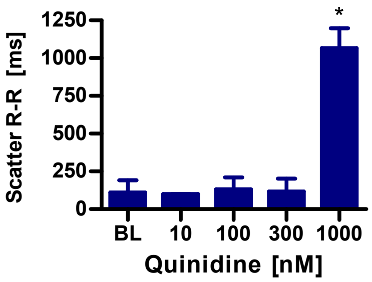

Scatter of beat-to-beat variability in the presence of quinidine, *$P<0.05$ (Mann-Whitney U test), 4 biological replicas, bars show median ± interquartile range.

Sertindole - original recordings

Sertindole baseline

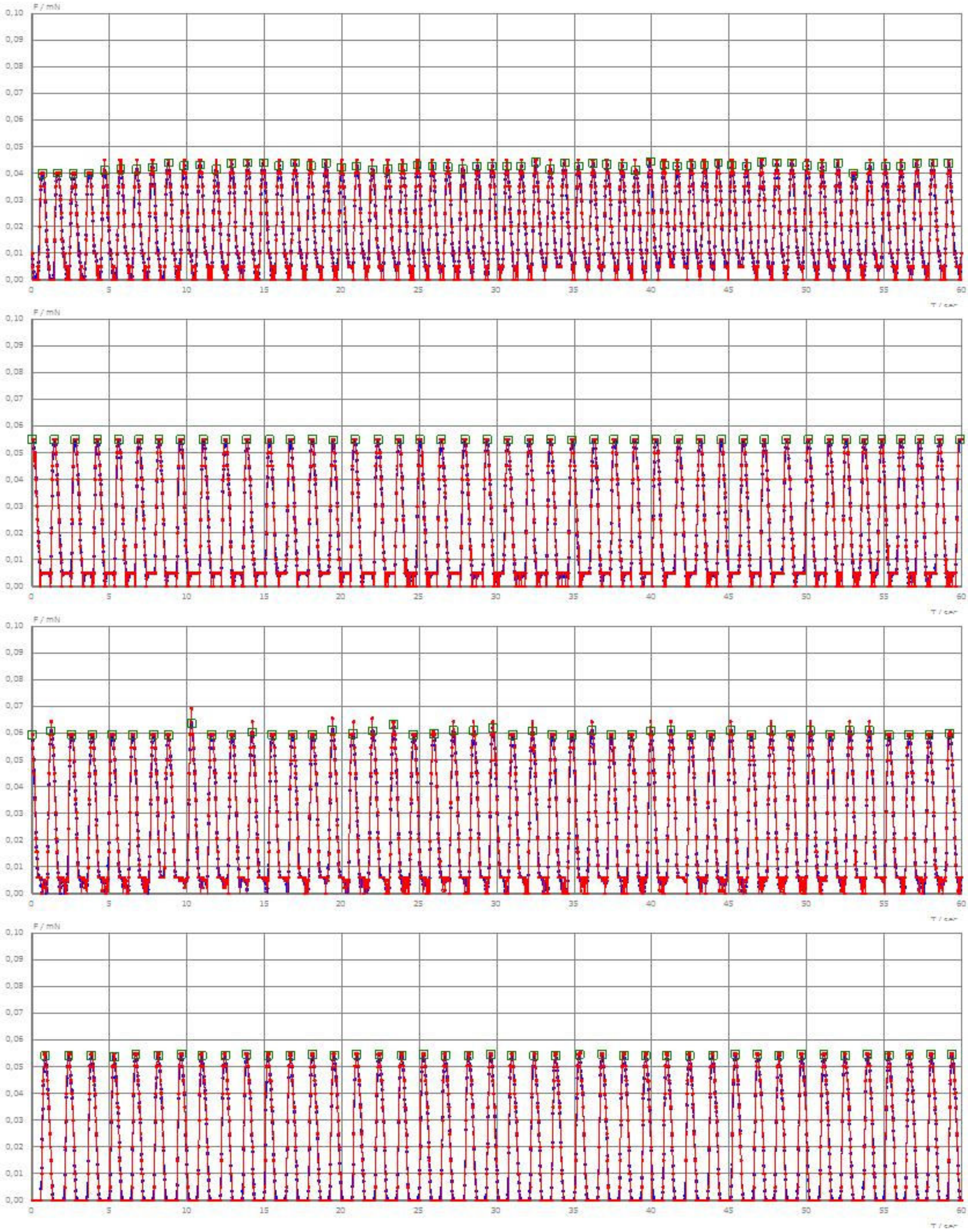

Sertindole 1 nM

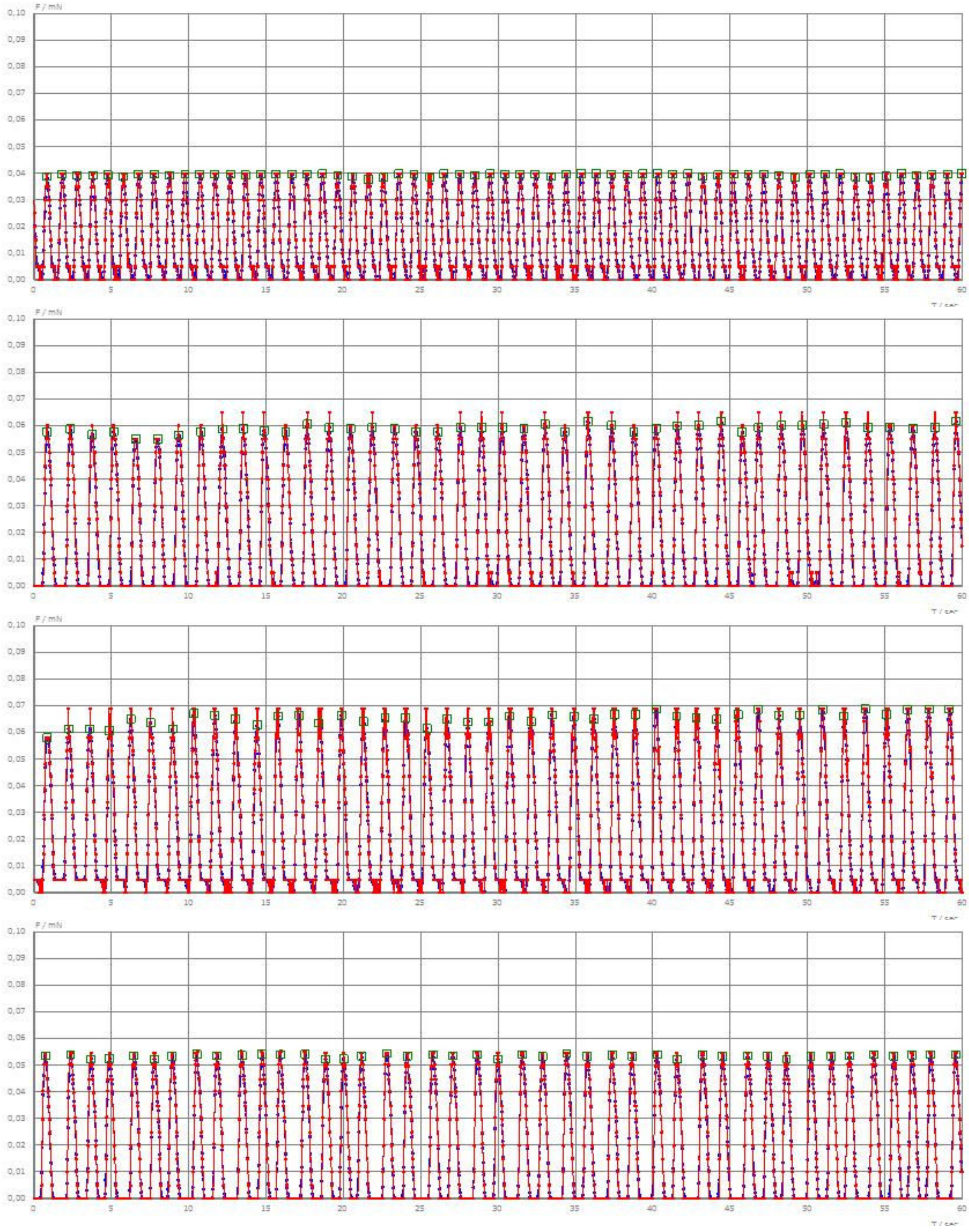

Sertindole 10 nM

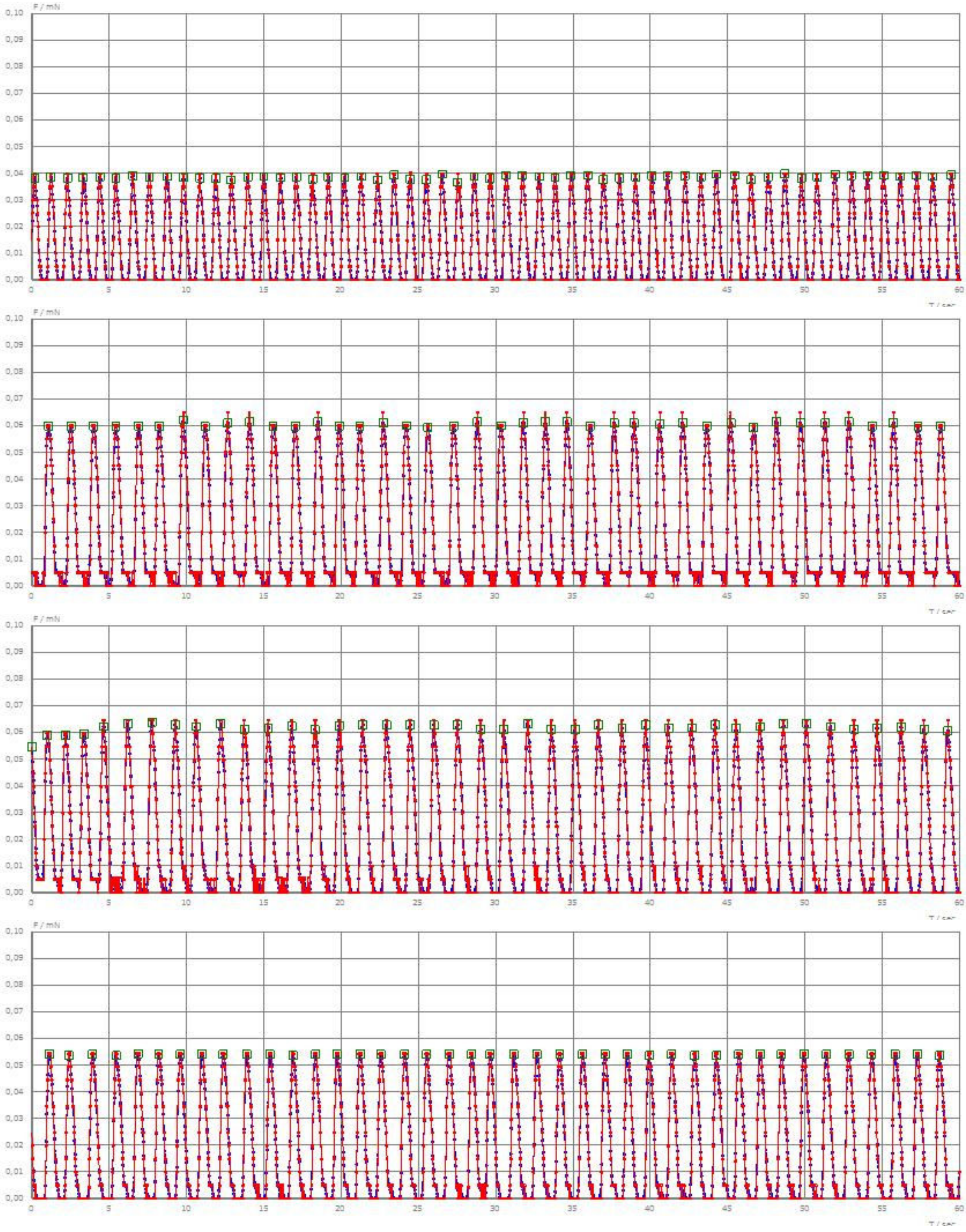

Sertindole 30 nM

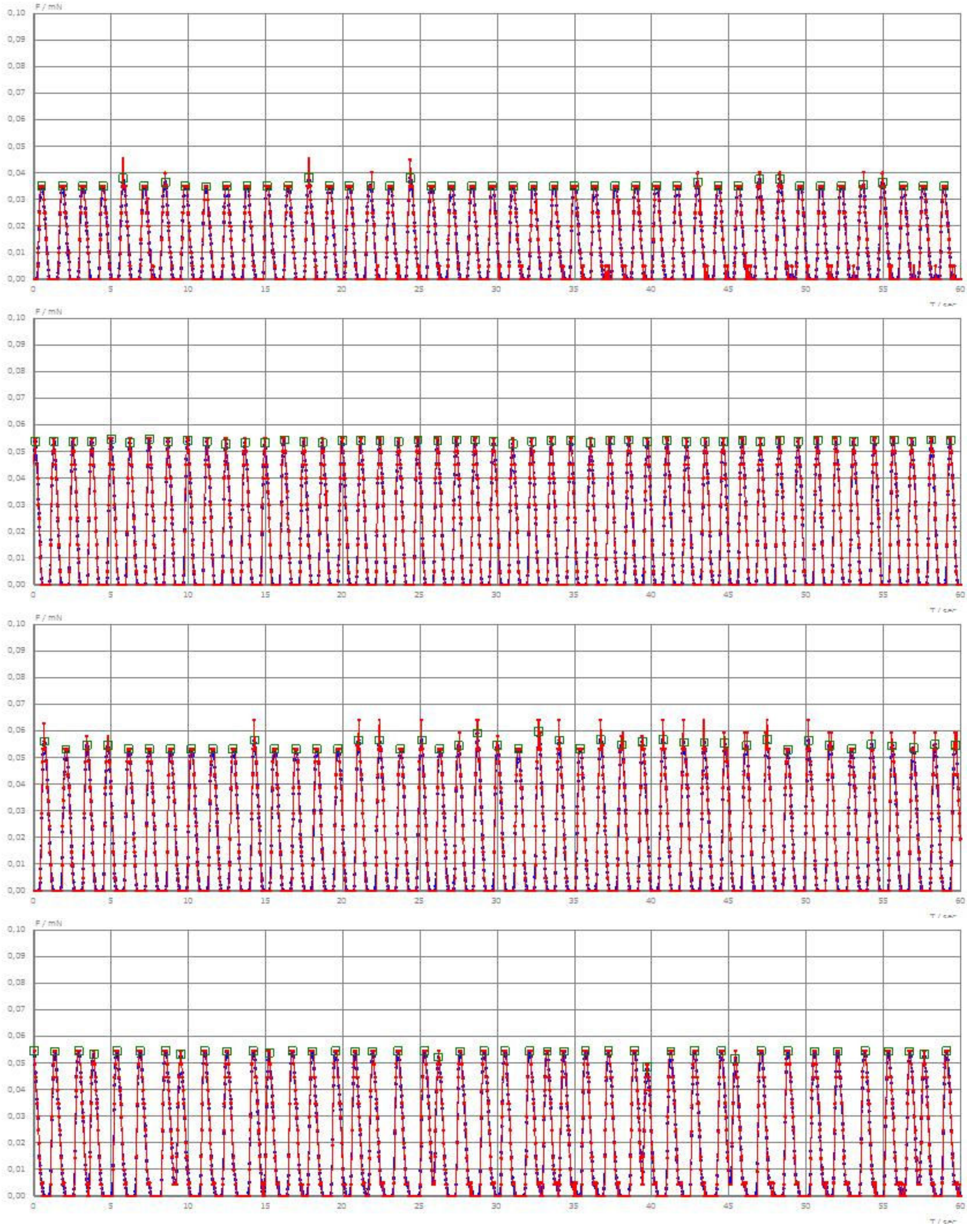

Sertindole 100 nM

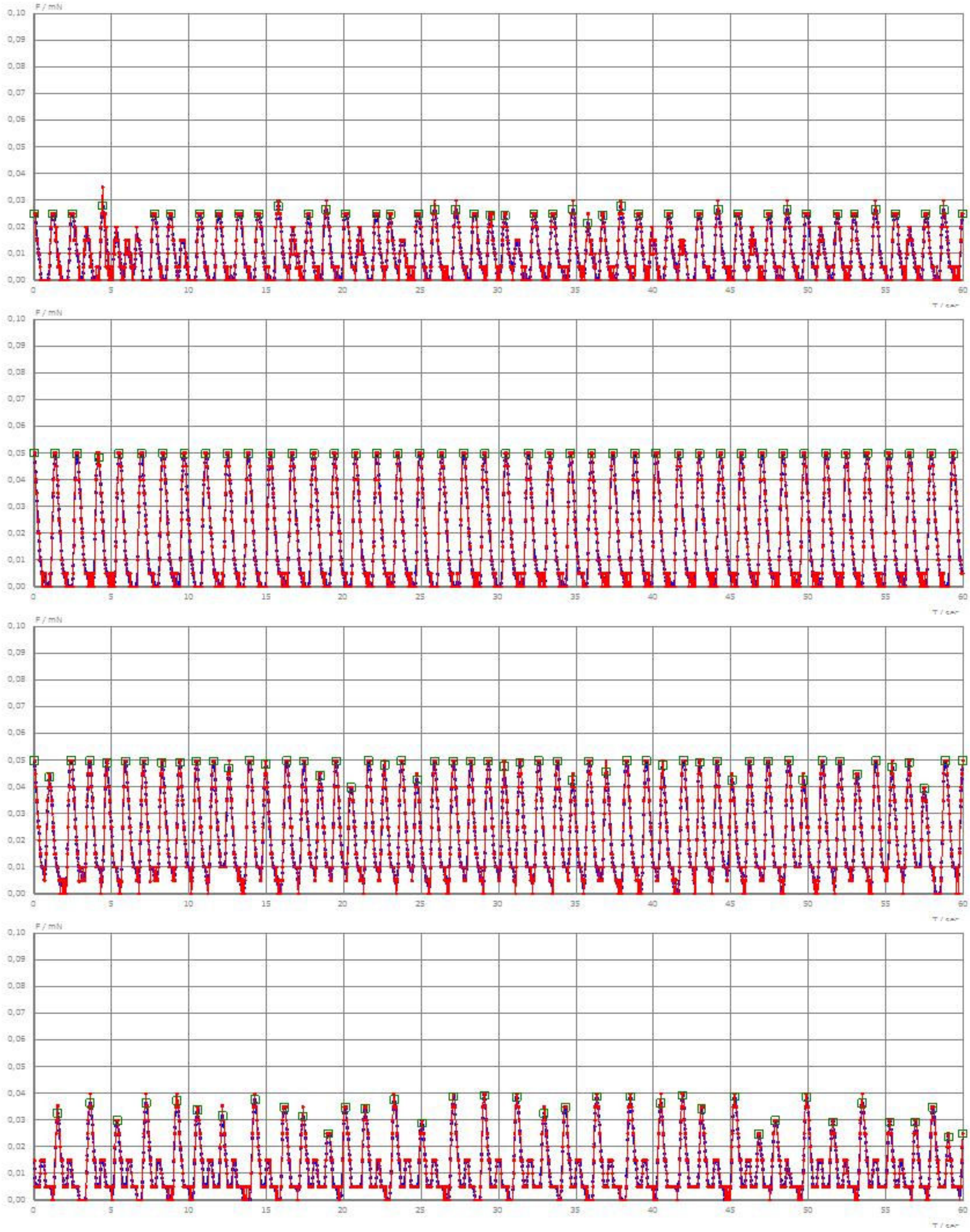

Sertindole 300 nM

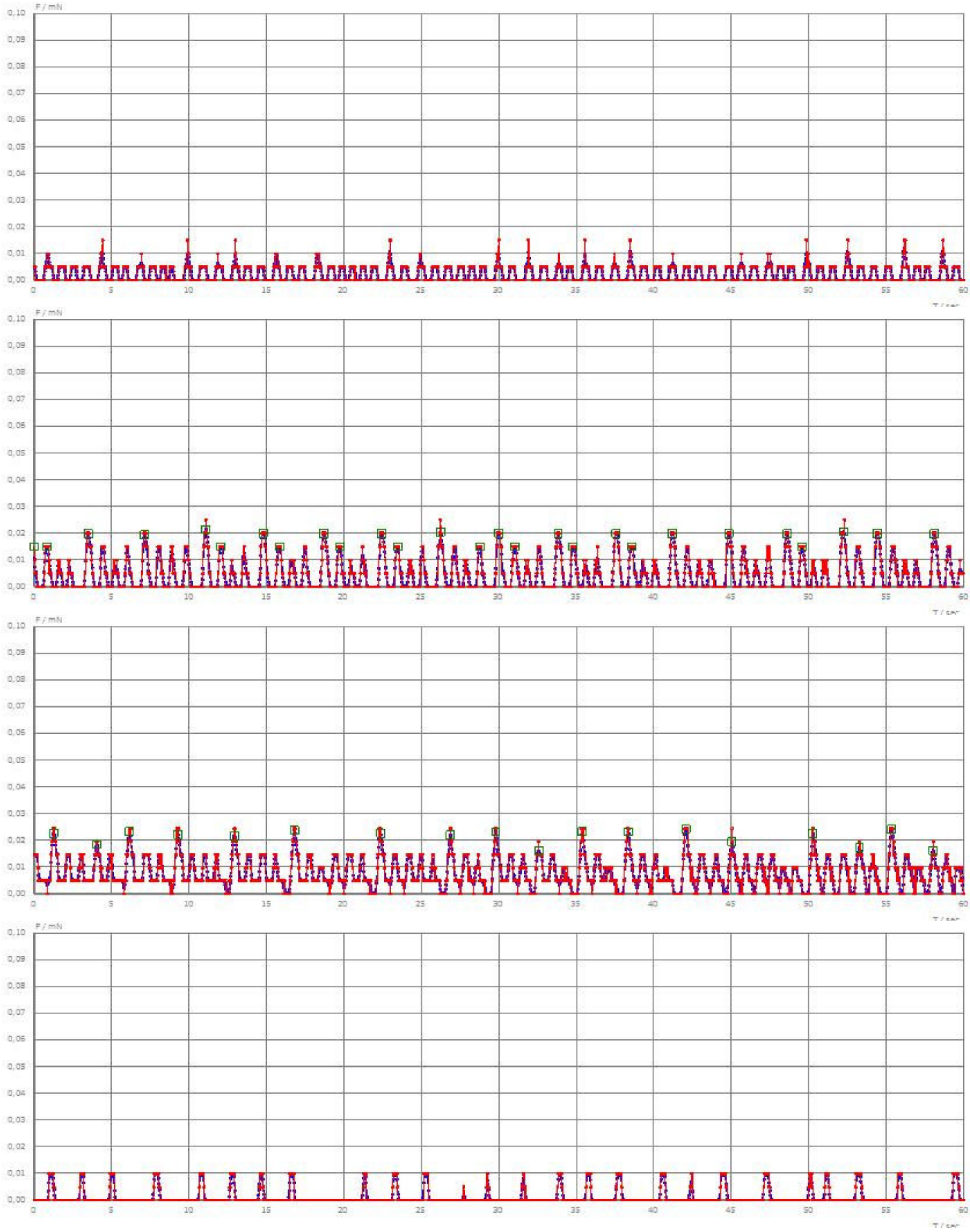

Analysis sertindole

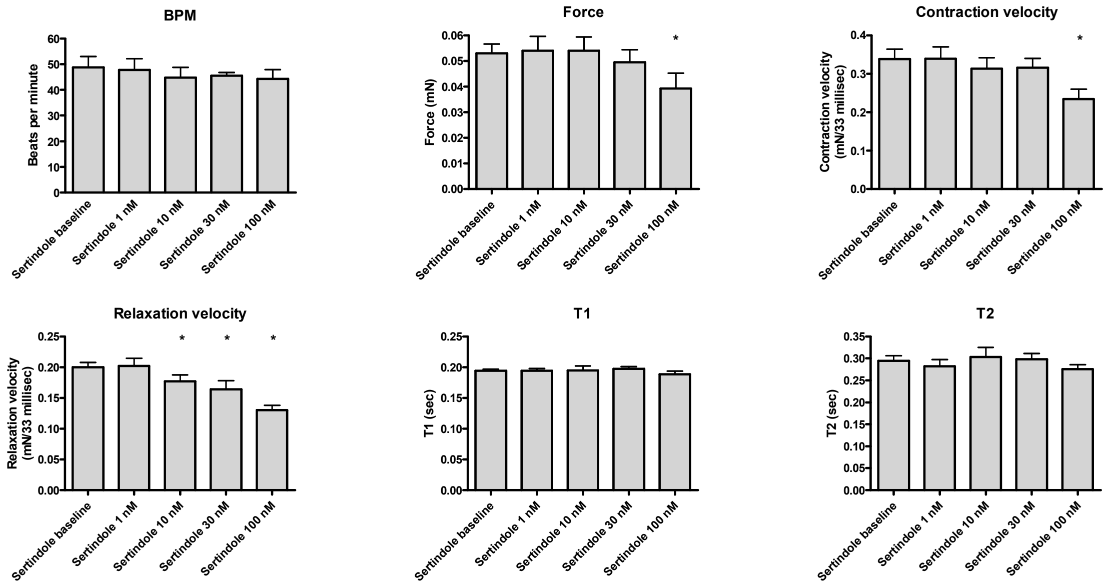

Analysis of contraction. **P*<0.05 (Student's t-test), 4 biological replicas, bars show means ± SD. Beats per minute (BPM), contraction time (T1), relaxation time (T2).

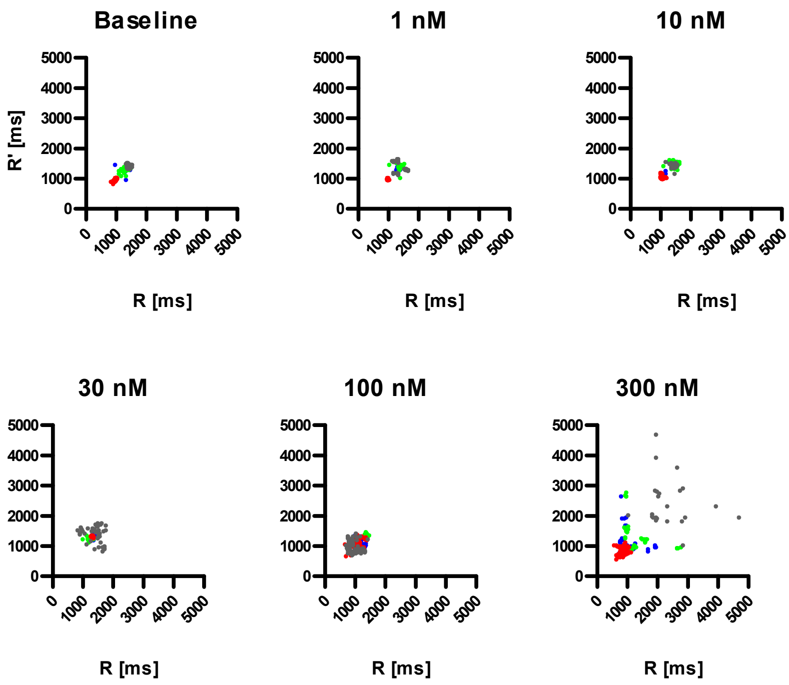

Graphical illustration of irregularity integrating 4 biological replicas. Ordinates indicate the distance from a given twitch to the following, the abscissa the distance to the previous twitch. Biological replicas are discriminated by color code.

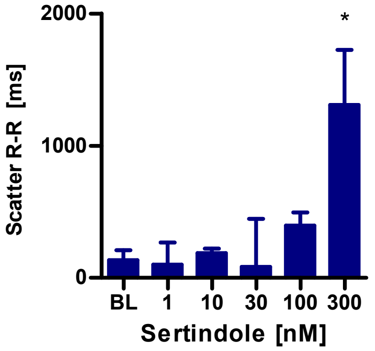

Scatter of beat-to-beat variability in the presence of sertindole, *P<0.05 (Mann-Whitney U test), 4 biological replicas, bars show median ± interquartile range.

Cisapride - original recordings

Cisapride baseline

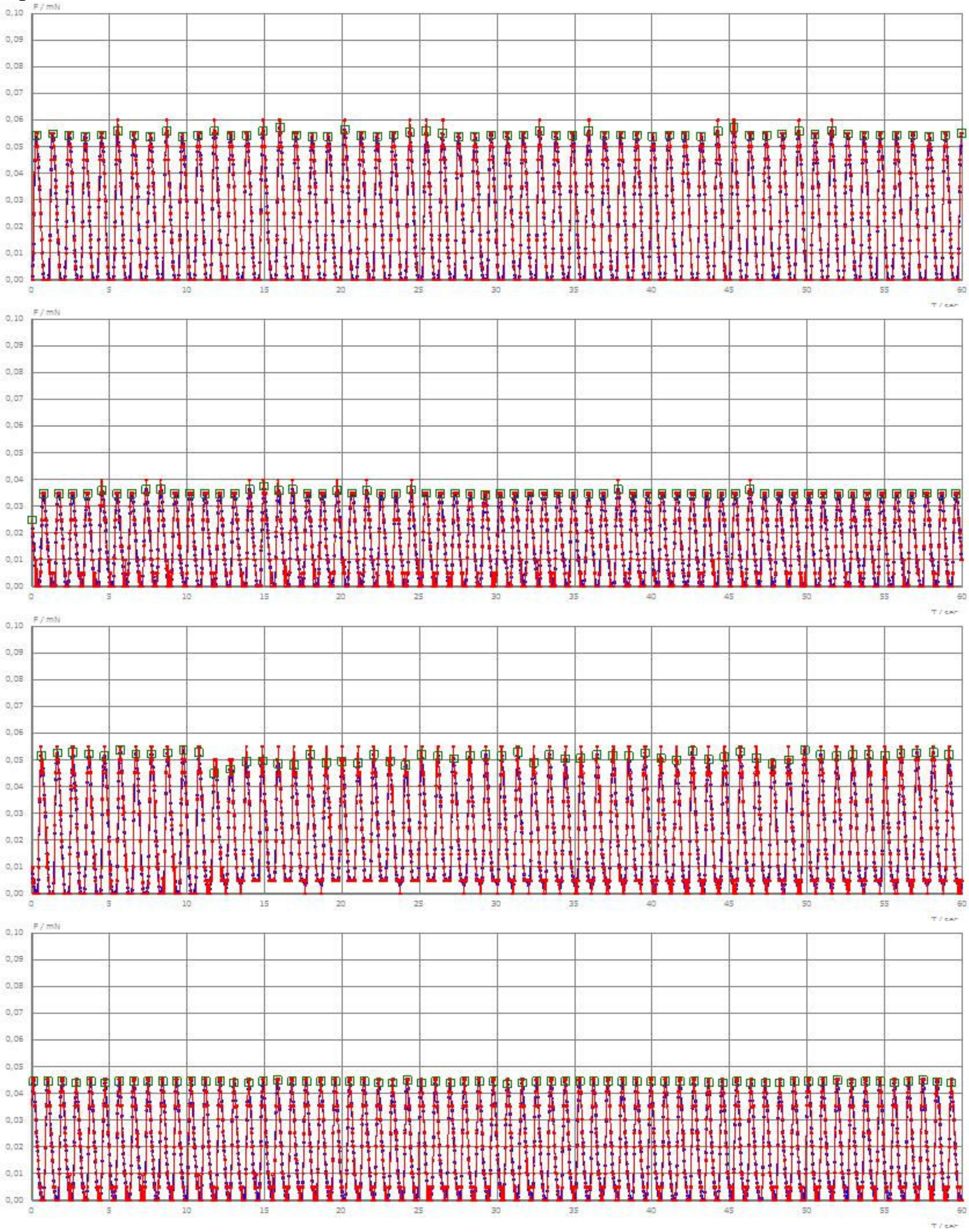

Cisapride 0.3 nM

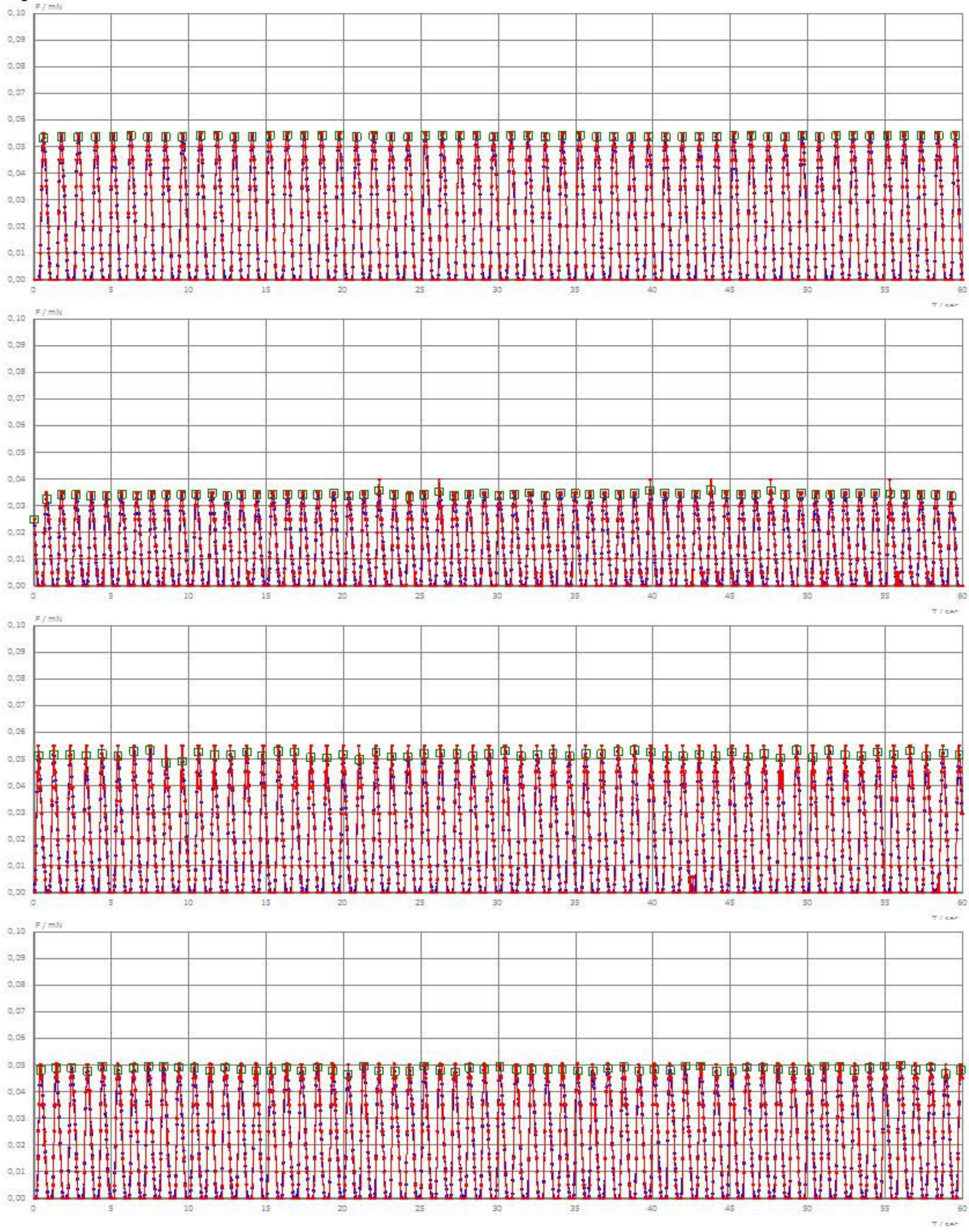

Cisapride 3 nM

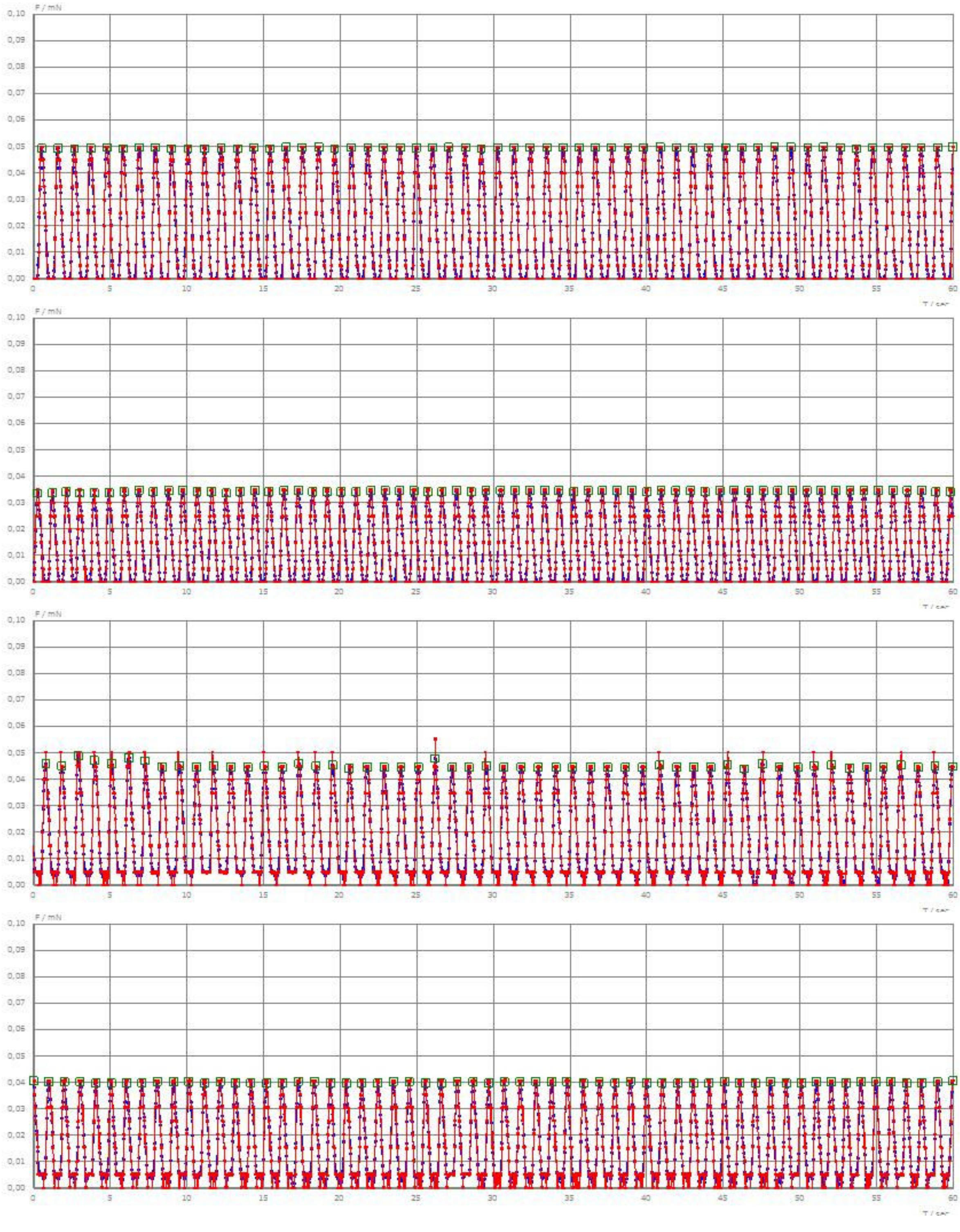

Cisapride 10 nM

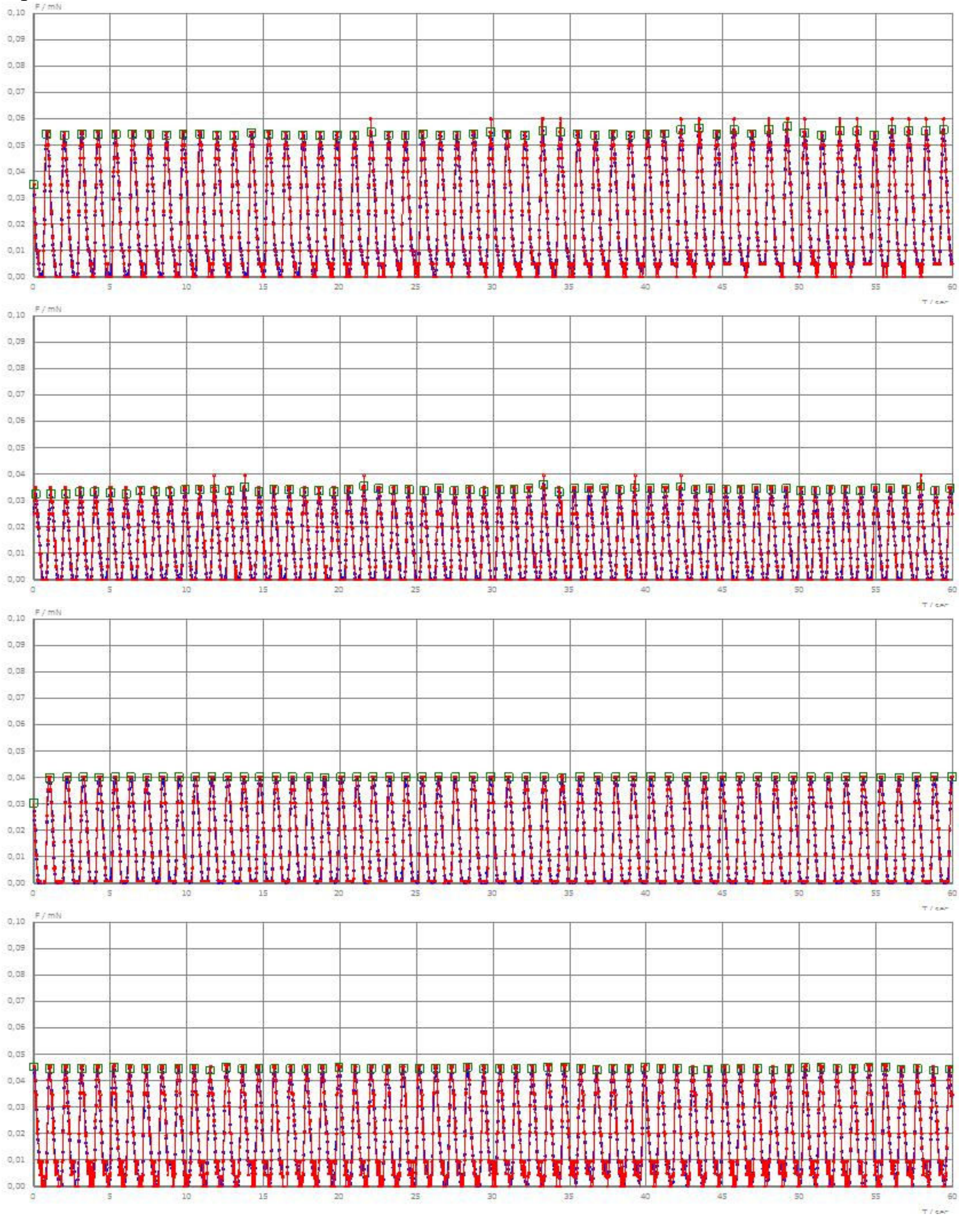

Cisapride 30 nM

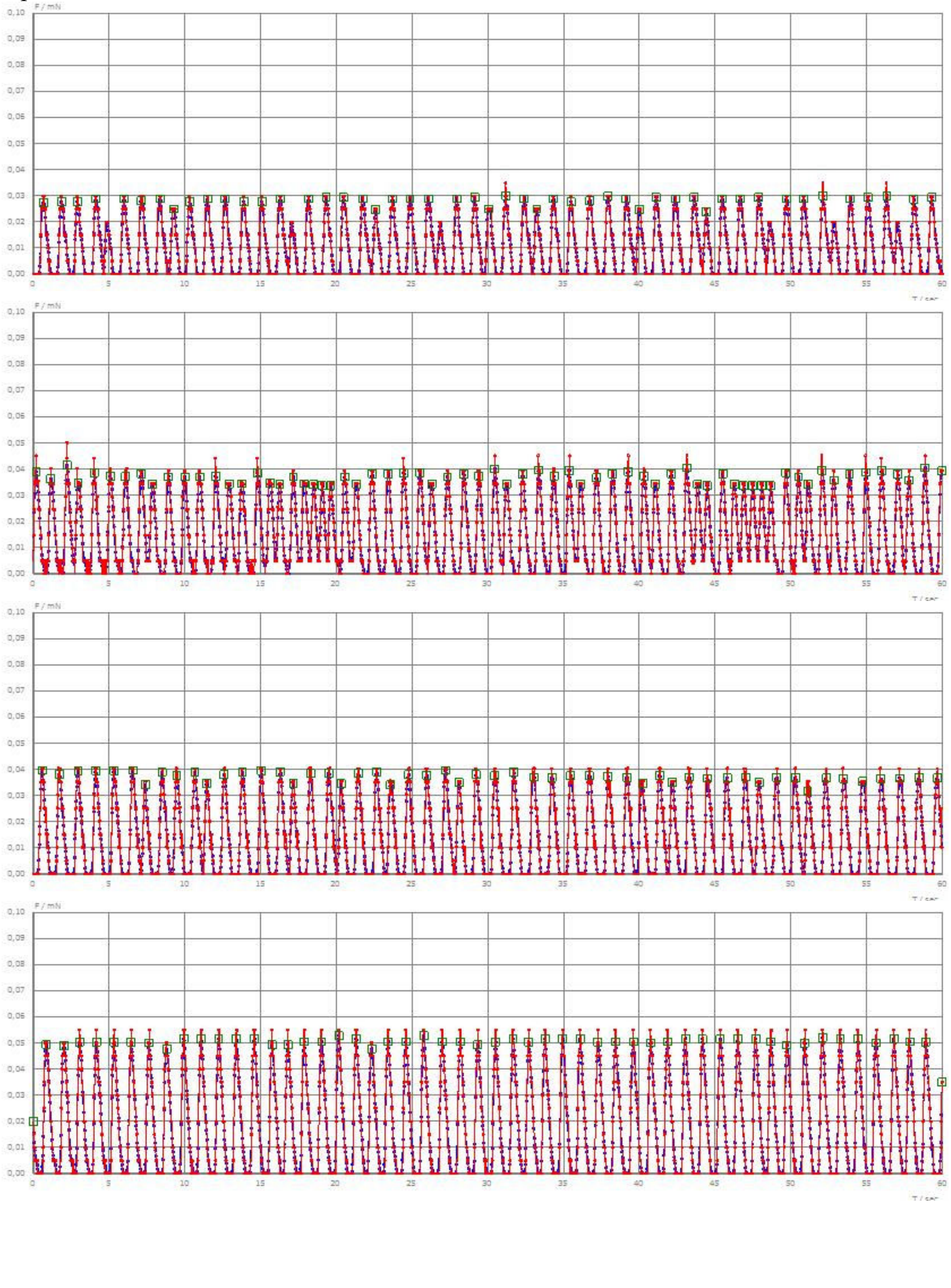

Cisapride 100 nM

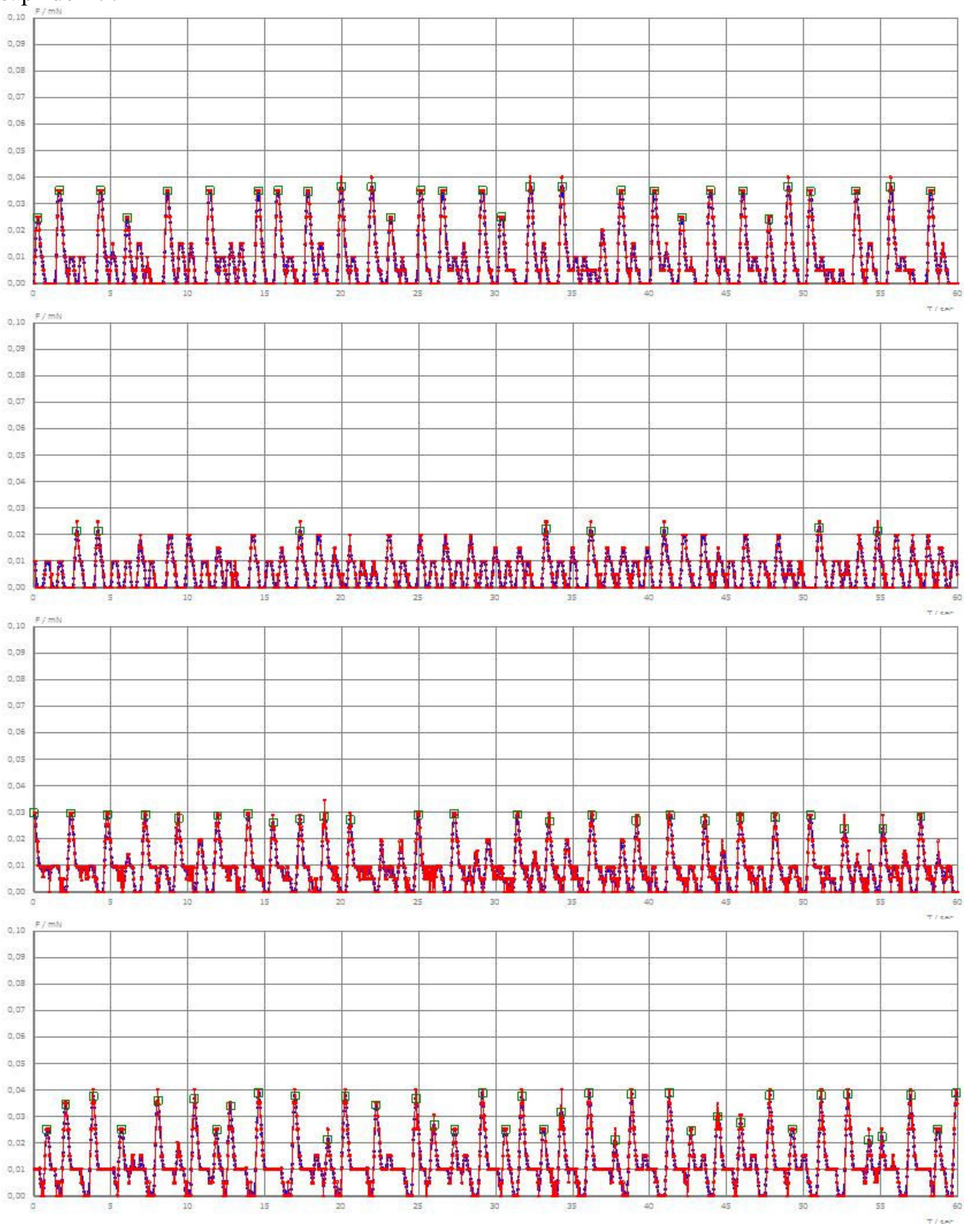

Analysis cisapride

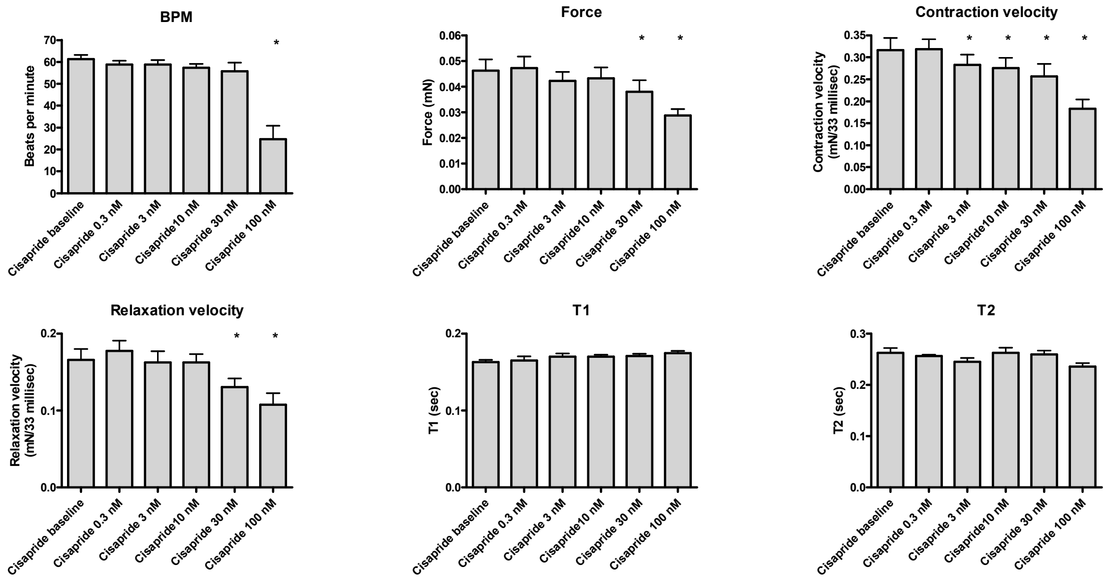

Analysis of contraction. **P*<0.05 (Student's t-test), 4 biological replicas, bars show means ± SD. Beats per minute (BPM), contraction time (T1), relaxation time (T2).

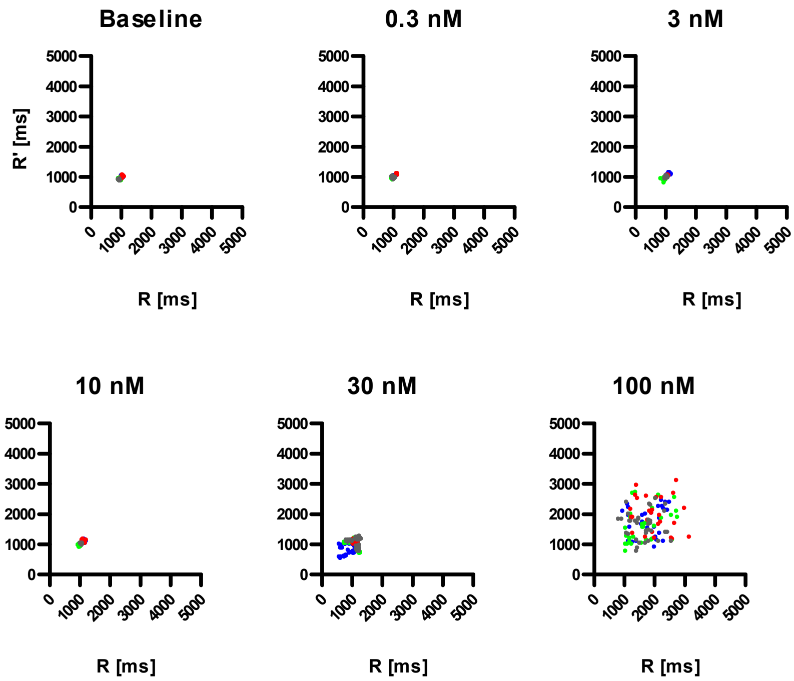

Graphical illustration of irregularity integrating 4 biological replicas. Ordinates indicate the distance from a given twitch to the following, the abscissa the distance to the previous twitch. Biological replicas are discriminated by color code.

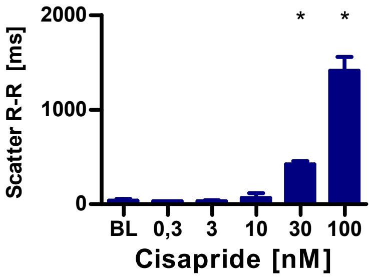

Scatter of beat-to-beat variability in the presence of cisapride, **P*<0.05 (Mann-Whitney U test), 4 biological replicas, bars show median ± interquartile range.

Ampicillin - original recordings

Ampicillin baseline

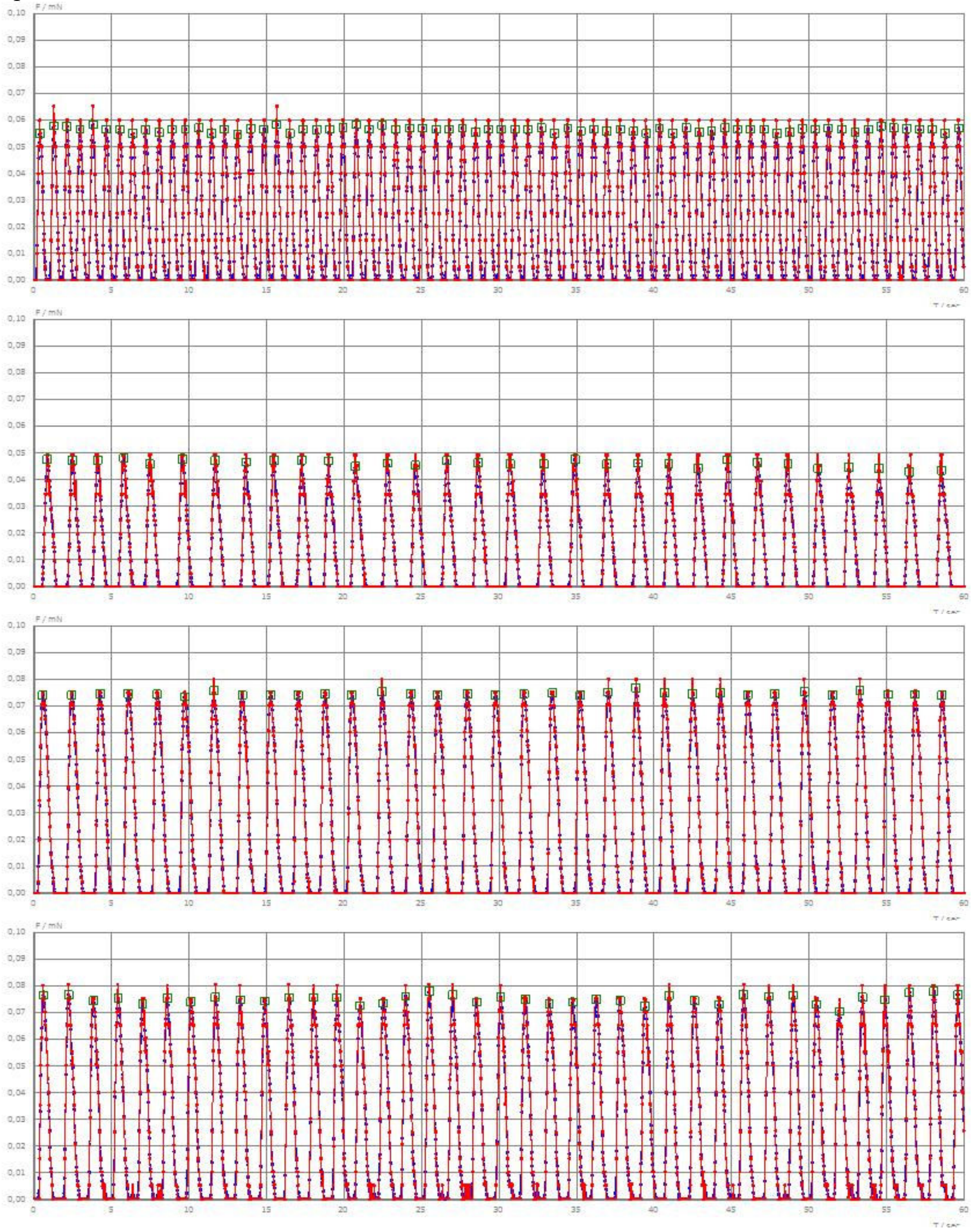

Ampicillin 10 µM

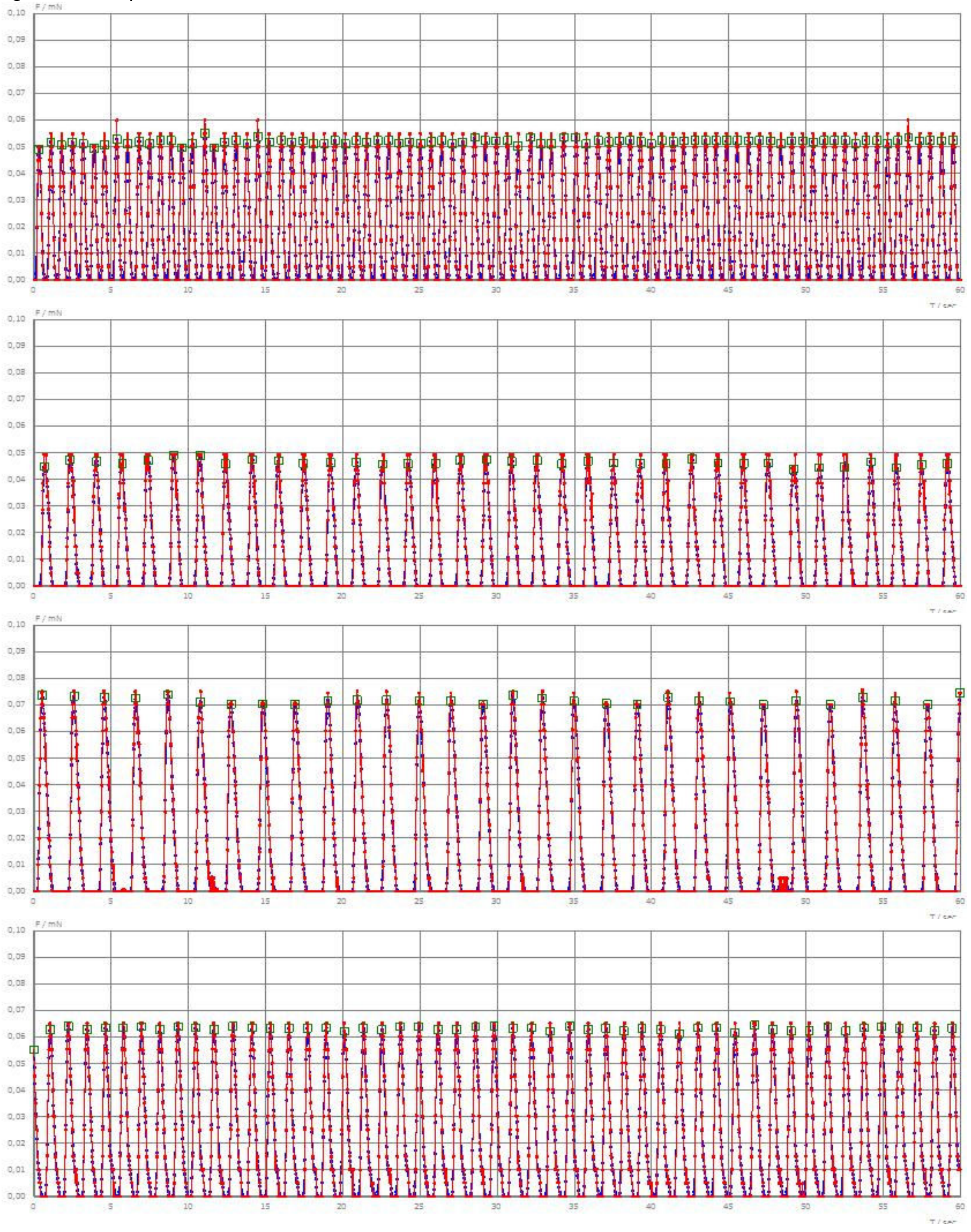

Ampicillin 100 μM

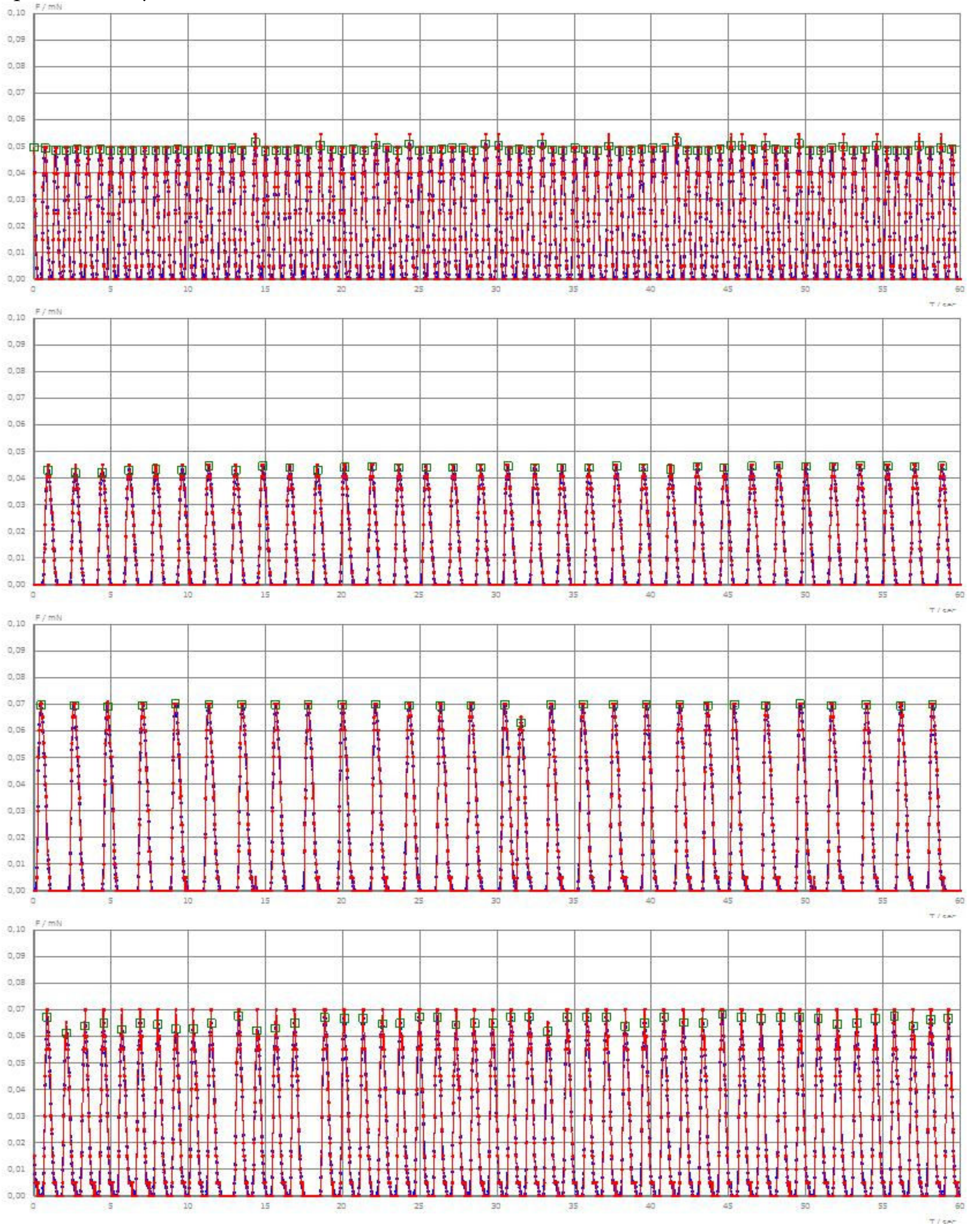

Ampicillin 1000 μM

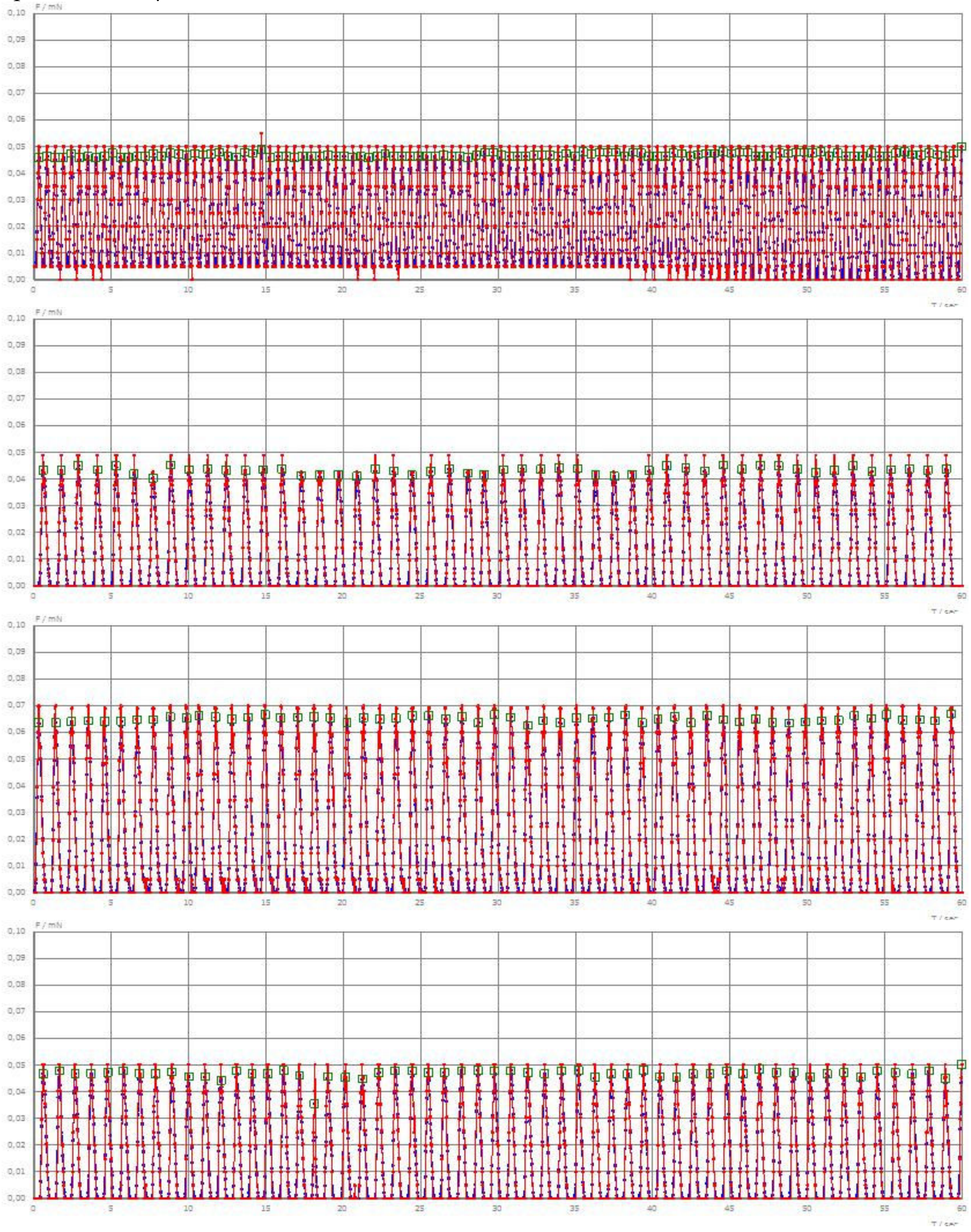

Analysis ampicillin

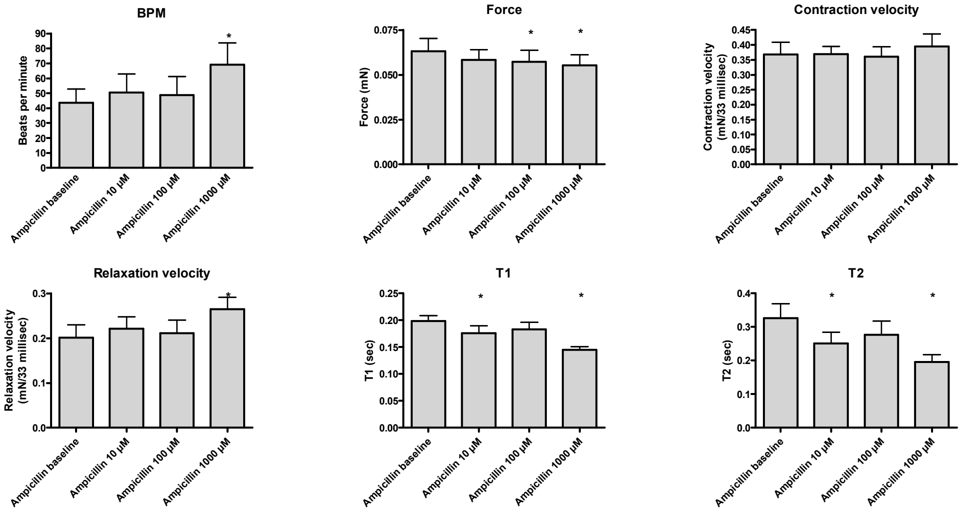

Analysis of contraction. *P<0.05 **(**Student's t-test**),** 4 biological replicas, bars show means ± SD. Beats per minute (BPM), contraction time (T1), relaxation time (T2).

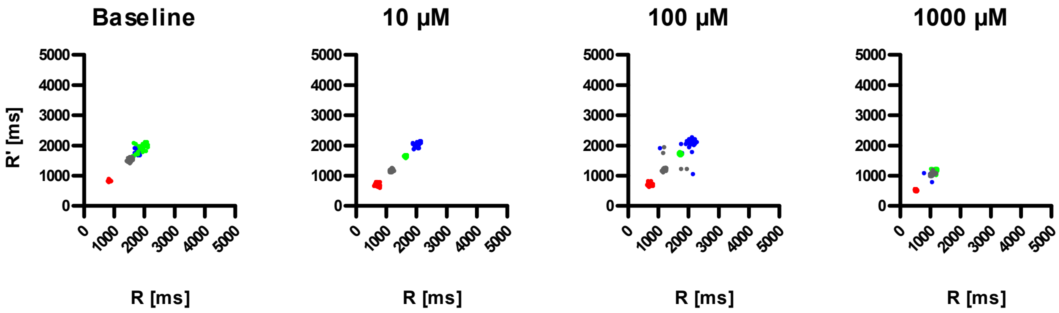

Graphical illustration of irregularity integrating 4 biological replicas. Ordinates indicate the distance from a given twitch to the following, the abscissa the distance to the previous twitch. Biological replicas are discriminated by color code.

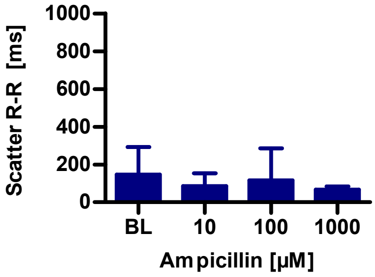

Scatter of beat-to-beat variability in the presence of ampicillin, *P<0.05 (Mann-Whitney U test), 4 biological replicas, bars show median ± interquartile range.
